# Unexpected link between polyketide synthase and calcium carbonate biomineralization

**DOI:** 10.1186/s40851-014-0001-0

**Published:** 2015-01-13

**Authors:** Motoki Hojo, Ai Omi, Gen Hamanaka, Kazutoshi Shindo, Atsuko Shimada, Mariko Kondo, Takanori Narita, Masato Kiyomoto, Yohei Katsuyama, Yasuo Ohnishi, Naoki Irie, Hiroyuki Takeda

**Affiliations:** Department of Biological Sciences, Graduate School of Science, University of Tokyo, 7-3-1 Hongo, Bunkyo-ku, Tokyo 113-0033 Japan; Tateyama Marine Laboratory, Marine and Coastal Research Center, Ochanomizu University, Kou-yatsu 11, Tateyama, Chiba 294-0301 Japan; Department of Food and Nutrition, Japan Women’s University, 2-8-1, Mejirodai, Bunkyo-ku, Tokyo 112-8681 Japan; Misaki Marine Biological Station, Graduate School of Science, University of Tokyo, 1024 Koajiro, Misaki, Miura, Kanagawa 238-0225 Japan; Department of Biotechnology, Graduate School of Agricultural and Life Sciences, University of Tokyo, 1-1-1, Yayoi, Bunkyo-ku, Tokyo 113-8657 Japan; Present address: Department of Pharmaceutical and Environmental Sciences, Tokyo Metropolitan Institute of Public Health, 3-24–1, Hyakunincho, Shinju-ku, Tokyo 169-0073 Japan; Present address: Division of Molecular Pathology, Research Institute for Biomedical Sciences, Tokyo University of Science, 2669 Yamazaki, Noda, Chiba 278-0022 Japan; Present address: Laboratory of Veterinary Biochemistry, Nihon University College of Bioresource Sciences, 1866 Kameino, Fujisawa, Kanagawa 252-0880 Japan

**Keywords:** Biomineralization, Calcium carbonate, Otolith, Polyketide synthase, Medaka, Spicule

## Abstract

**Introduction:**

Calcium carbonate biominerals participate in diverse physiological functions. Despite intensive studies, little is known about how mineralization is initiated in organisms.

**Results:**

We analyzed the medaka spontaneous mutant, *ha*, defective in otolith (calcareous ear stone) formation. *ha* lacks a trigger for otolith mineralization, and the causative gene was found to encode polyketide synthase (pks), a multifunctional enzyme mainly found in bacteria, fungi, and plant. Subsequent experiments demonstrate that the products of medaka PKS, most likely polyketides or their derivatives, act as nucleation facilitators in otolith mineralization. The generality of this novel PKS function is supported by the essential role of echinoderm PKS in calcareous skeleton formation together with the presence of PKSs in a much wider range of animals from coral to vertebrates.

**Conclusion:**

The present study first links PKS to biomineralization and provides a genetic cue for biogeochemistry of carbon and calcium cycles.

**Electronic supplementary material:**

The online version of this article (doi:10.1186/s40851-014-0001-0) contains supplementary material, which is available to authorized users.

## Introduction

Biominerals are produced by living organisms through genetically controlled biological processes, and confer stability, rigidity, defences and functionality to organisms; examples include grass opal (rice; silicate), shell (shellfish; calcium carbonate), and bone/teeth (vertebrate; hydroxyl apatite) [1,2]. The addition of organic components determines organism-specific shape, size, hardness and crystallographic axis orientation of biominerals [3]. Among biominerals, calcium carbonate is the most abundant and is known to play an important role in the global biogeochemical cycles of carbon and calcium through reef building and massive precipitation by calcifying algae in the ocean [4-6], and thus has attracted much attention, together with calcium phosphate mineralization of vertebrate skeleton and teeth.

The biomineral crystallization begins with nucleation through the phase of prenucleation clusters [7,8]. Experimental and modeling studies proposed that nucleation from saturated solution is not a simple event, but is preceded by a phase of amorphous precursors, such as amorphous calcium carbonate (ACC) or amorphous calcium phosphate, depending on the type of biominerals. These precursors then coalesce and rearrange to form nuclei, a core of subsequent crystal growth, a process called ‘nucleation’. Although these processes of biomineralization are well described, the underlying mechanism, particularly regarding the initial nucleation step, remains a mystery.

Otoliths (ear stones) in teleost fish are composed of calcium carbonate and organic materials (0.2–10%) [9], and primarily function in gravity and motion sensing, providing excellent systems for biochemical and genetic analyses of the mechanisms of calcium carbonate biomineralization. Factors involved in initial step of mineralization have not been definitively identified thus far, except for a report that glycogens are always found in the core of nascent otoliths [10]. This is in part due to the lack of genetic studies targeting otolith mineralization. Indeed, while most zebrafish otolith mutants exhibit defects in the number, shape, and position, only a few completely lack otoliths, and in the cases where the causal gene was identified, its function in the context of mineralization remained unclear [11,12].

To gain insights into the crystallization of calcium carbonate in organisms, we analyzed the medaka spontaneous and homozygous viable mutant, *ha*, defective in otolith formation. Positional cloning identified a mutation in a novel gene encoding polyketide synthase (pks), named OlPKS, specifically expressed in the otic vesicle at the onset of mineralization. We demonstrate that the compound synthesized by OlPKS is secreted in the endolymph and it acts as a nucleation facilitator in otolith mineralization. Furthermore, sea urchin *pks-2* expressed in skeletogenic cells was found to be indispensable for the formation of spicules, calcareous larval skeletons, indicating the generality of a novel link between calcium carbonate biomineralization and animal polyketide synthase.

## Materials and methods

### Fish strains and mapping

The medaka (*Oryzias latipes*) d-rR strain was used as a wild-type. *ha* was isolated as a spontaneous mutant by Tomita [13]. *ki79* was isolated from a ENU-mediated mutant screening (at the Tokyo Institute of Technology, Japan). Mapping of the *ha* locus was carried out as previously described [14].

### Otolith staining

Alizarin Red staining for visualizing mineralized otoliths was essentially done as described [15]. Embryos were fixed with 4% PFA/2× PBS (phosphate buffered saline) containing 1% sodium hydroxide for three hours at room temperature. The samples were then washed with PBS several times, and stained with 2% Alizarin Red S/1% sodium hydroxide solution overnight. After quickly washing with 0.5% KOH, samples were mounted in 80% glycerol.

### Transmitted electron microscopy

Embryos were fixed with 2% PFA and 2% glutaraldehyde buffered in 0.1 M cacodylate buffer and postfixed in 2% OsO_4_ in 0.1 M cacodylate buffer. Uranyl acetate and lead stain solution were used for contrast enhancement. The specimens were embedded in Quetol-812. Ultrathin sections (70 nm) were analyzed and documented with an electron microscope (JEOL, JEM-1400 Plus).

### Whole-mount *in situ* hybridization

All whole-mount *in situ* hybridization analyses were performed as previously described [16]. cDNAs isolated by RT-PCR were used as the templates for the probes of marker genes (see Additional file 1).

### Immunofluorescence

Immunofluorescence for OMP-1 was performed as previously described Murayama *et al.* [17], whereas staining for acetylated α-tubulin, OlPKS and PKC ζ were done as previously described by Kamura *et al.* [18] (see Additional file 1). Polyclonal rabbit OlPKS [amino acids 1520–1711] antibodies were raised by immunization of rabbits with bacterially expressed His-tagged truncated proteins.

### Morpholino knockdown and mRNA rescue in medaka embryos

The morpholino antisense oligonucleotide (MOs; Gene Tools LLC) for first-Met of OlPKS was as follows: 5'-AACCACGGCTATTCCGTCCTCCATG-3'. *in vitro* syntheses of mRNAs were conducted as reported previously [19]. Full length of *olpks* was isolated from the d-rR strain by RT-PCR. The sequences of primers are described in Additional file 1 (Whole-mount *in situ* hybridization). For injection of *olpks* mRNA with active site mutation, *ki79* fish was used, because it likely carries a null mutation. Amino acid of the active site in each enzymatic domain was substituted as followed (with nucleotide sequences): KS, 173Cys → Asn (TGC → AAC); AT, 608Ser → Ala (TCC → GCA); DH,914His → Ala (CAC → GCC); KR,1859Tyr → Phe(TAC → TTC); ACP, 2010Ser → Ala(TCC → GCC); Loop (interdomain region; control), 435Thr → Ala(ACC → GCC) [20-22]. Microinjection of MO or mRNAs was carried out as previously described [19].

### Genomic research

Animal type I PKS sequences were retrieved by TBLASTN against genome sequences of each species or BLASTP search against nr-protein database using the OlPKS sequence. For each candidates, domain search was done by Pfam sequence search, and amino acid sequences having basic PKS domains KS and AT, and additional domains (*e.g.*, DH, KR, ER, ACP etc.) were defined as an animal type I PKS.

### Generation the *ha* chimeric medaka

Transplantation experiments were performed based on the previous works [23]. [Tg (β-actin:DsRed)] were used as *wt*-cell donors, whereas *ha* fish were prepared as hosts. Whether otoliths were formed or not and how many fluorescence cell were contained were confirmed by observations around st. 29 using a confocal fluorescence microscope (Zeiss, LSM710). A transplantation experiment was conducted as a negative control in which [*ha*^−/−^; Tg (β-actin:DsRed)] animal was used as the donors (detailed in Additional file 1).

### OlPKS expression in *A. oryzae* and medaka rescue assay

The entire OlPKS ORF was amplified and subcloned into a fungal expression vector, pTAex3. The resulting expression plasmid pTA-*olpks* was used for transformation of *A. oryzae* M-2-3 according to the protoplast-PEG method described by Gomi *et al.* [24]. The mycelia of transformants were extracted with acetone, followed purification using partition between ethyl acetate/H_2_O two times and the ethyl acetate layer was concentrated to dryness, and partitioned between hexane/90% methanol. The methanol layer was concentrated to dryness to give the material for medaka assay. Each extracts re-dissolved in DMSO was added to the medium in which *ha* medaka embryos were incubated. (detailed in Additional file 1).

### Morpholino knockdown in sea urchin embryos

Microinjection of MOs was carried out as described previously with some modifications [25] (see Additional file 1). MOs (Gene Tools LLC) for first-Met blocking and their five-mispair controls were as follows (small letters in control sequences indicate substituted nucleotides).

*hppks-1*: 5'-CTGGTTTTATTGCTTCCCATGTTGA-3', *hppks-2*: 5'-CCCTCCAACTATCTTCCATAACTCA-3', *hppks-1 control*: 5'- CTGcTaTTATTcCTTCCgATcTTGA -3', *hppks-2 control*: 5'-CCgTgCAAgTATgTTCgATAACTCA-3'.

## Results

### The *ha* embryo fails to mineralize otoliths

**Figure 1:**
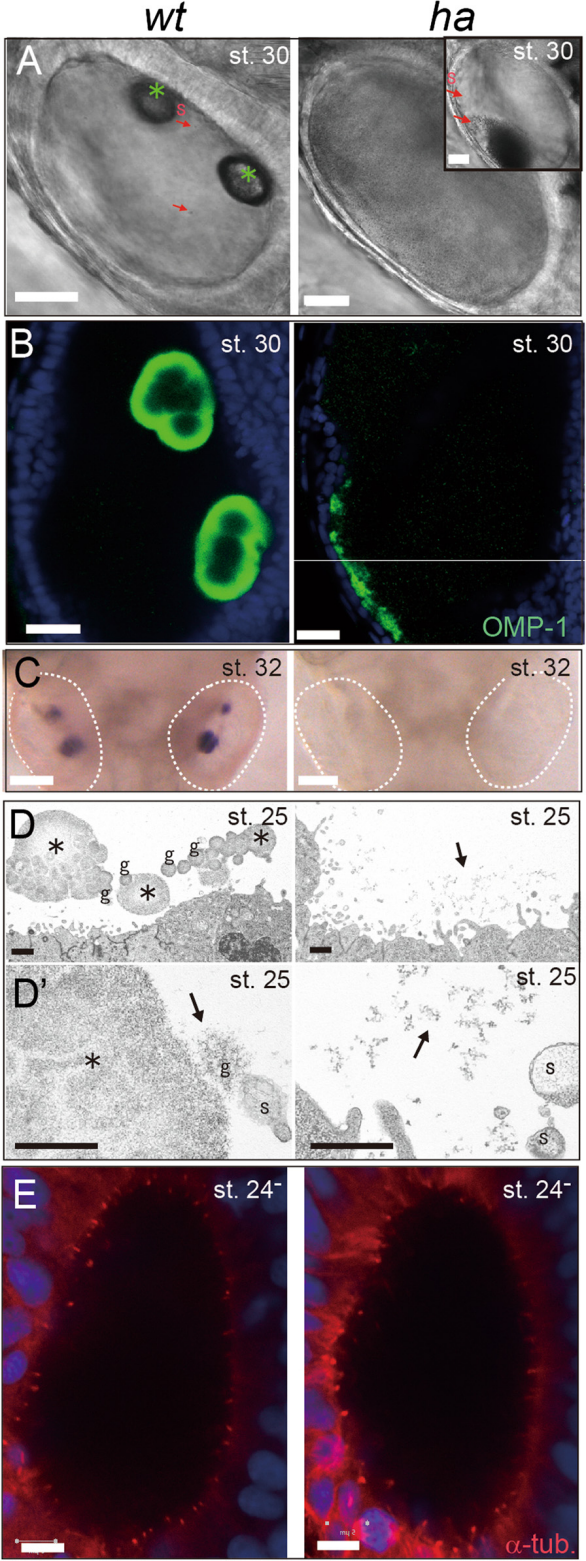
***ha*** **embryos fail to mineralize otoliths. (A)** DIC images of OVs at st. 30 (dorsal views of the left OV). Grown otoliths are observed in *wt* OV. Mutant OV contains numerous seeding particles that form a paste-like precipitate (*Inset)*. Red arrows: seeding particles; Asterisks: otoliths. Scale bars: 20 μm. **(B)** Immunofluorescence of an otolith matrix protein, OMP-1 (dorsal views of the left OV). Anti-*Oncorhynchus mykiss* (Om) OMP-1 serum is used. In mutant OV, immunoreactive substances cling to the epithelium. Scale bars: 20 μm. **(C)** Alizarin Red staining for mineralized otolith. Crystal is never observed in mutant OV (dorsal views of the head; white dotted lines show OV). Scale bars: 100 μm. **(D)** TEM images of the epithelium of the OV at st. 25 when the otolith is forming (the prospective macula region; lateral views). In a *wt* embryo ‘globules’ coalesce to form the otolith precursor in the posterior end of the OV. In the mutant, by contrast, very fine particles are observed at posterior end of OV (D) and mid-position of the OV (D’). Asterisks: growing otoliths; Black arrows: fine particles; ‘g’:globule; ‘s’:seeding particle. Scale bars: 1 μm. **(E)** Immunofluorescence of acetylated α-tubulin st. 24^−^ (dorsal views of the left OV). Many short cilia protruded from the epithelium are visible in *ha* OV as well as *wt* one. Scale bars: 5 μm.

Medaka *ha* is a spontaneous and homo-viable mutant defective in otolith formation [13] (Figure 1A and C). The gross morphology of *ha* was previously reported as significantly delayed mineralization of otoliths, slightly enlarged otic vesicles (OVs) and malformed semicircular canals [26,27]. We further characterized the *ha* phenotypes using molecular markers at embryonic stages at which the formation of the OV and otoliths normally takes place (st. 22– 30).

**Figure 2:**
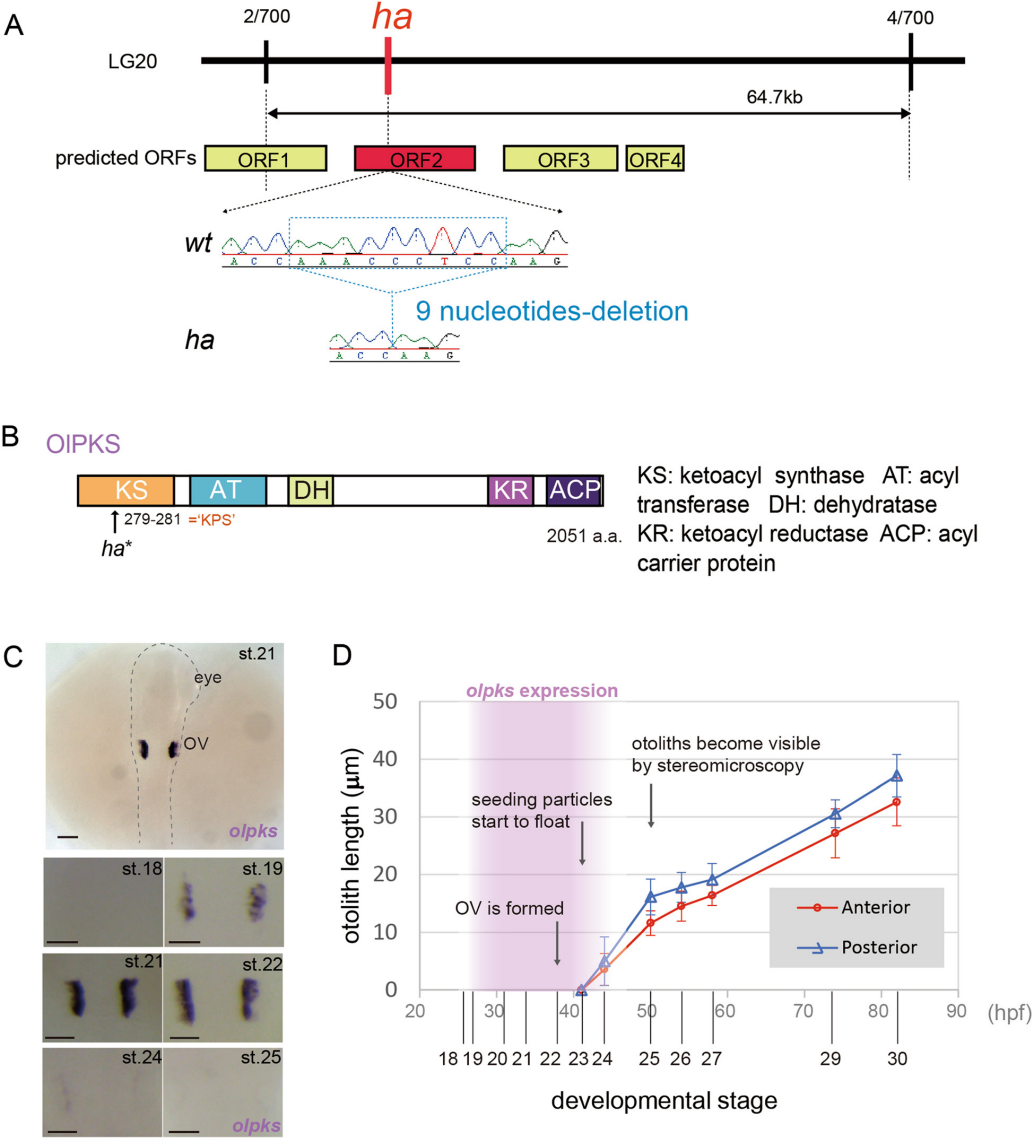
***ha*** **gene encodes a polyketide synthase. (A)** Positional cloning of the *ha* mutation in linkage group (LG) 20. The number of recombinants at each marker is shown. Sequencing of *ha* revealed a 9-nucleotide deletion. ORF: open reading frame. **(B)** Architecture of OlPKS (2051 amino acid-length) predicted by a Pfam search. Each domain is shown by abbreviation. An arrow indicates mutation site of *ha*, which is located at 279–281 (K, P and S). **(C)** Whole-mount *in situ* hybridization with an antisense RNA probe for *olpks* at otolith forming developmental stages. A representative picture is shown at st. 21 (*Upper*; dorsal view; dotted line indicates embryonic body). *olpks* transcripts detected in various stages are shown at high magnification (*Lower*; dorsal views of left and right OVs). Scale bars: 50 μm. **(D)** Period of the expression of *olpks* in the context of otolith growth. Purple area shows the period of *olpks* expression. Line graphs show the sizes (longest linear dimensions) of otoliths at some developmental stages. Data are the means and standard deviations of measurements taken of at least 7 specimens each. Some observable changes in the OV during otolith development are described with arrows. Red curcle: anterior otolith; Blue triangle: posterior otolith; hpf: hours post fertilization.

Otolith formation in medaka proceeds in a way similar to zebrafish [28]; ‘seeding particles’ start to float in the endolymph of the OV (Figure 1A and D) from st. 23 and coalesce into small crystal at st. 24 (Additional file 2K; *Upper Left*). Otoliths are stereo-microscopically visible as two small crystals at st. 25 and continuously increase in size (Figure 1A and D, and Figure 2D). In zebrafish, essential roles of cilia in otolith formation have been repeatedly shown [28-31]; tethering seeding particles by long kinocilia (5–8 μm) protruded from hair cells in the prospective macula regions and stirring the fluid by shorter motile cilia (1.5–5 μm) lining the entire of the OV epithelium. However, this scenario may not hold true in medaka embryos. Though cilia were found on the OV epithelium of medaka, they are much smaller in size (<1 μm) and their motility was hard to be detected (Figure 1E). Furthermore, probably due to their small size, we failed to identify kinocilia. At least, motile cilia do not contribute to otolith formation in medaka, as the medaka mutant *kintoun* with paralyzed cilia develops otoliths as normal (Additional file 2K) [32].

In *ha* mutant embryos, the OV, macula and cilia develop normally (Additional file 2A to D, I and J), and seeding particles are floating in the OV (Figure 1A and Additional files 3 and 4). Furthermore, organic materials such as OMP-1, a major soluble organic matrix protein, are secreted into the endolymph (Figure 1B) (other known organic components, *starmaker-like* and *sparc* are normally expressed in the OV (Additional file 2E and F)). Nevertheless, in *ha* mutants, mineralized stones never form (Figure 1A and C), but instead OMP-1-positive particles precipitate in the endolymph (Figure 1A *Inset* and B). This was further supported by TEM observation; fine substances (Figure 1D and D′, arrows), probably organic substances supplied by seeding particles, were accumulated around the epithelium in *ha* (Figure 1D *Right* and 1D’ *Right*), instead of growing otoliths (Figure 1D *Left* and D′ *Left*, asterisks). In wild-type (*wt*) OVs, fine particles were compacted into a round ‘globule’ (Figure 1D *Left*, ‘g’; [10]), and the globules then form otoliths by coalescing near the macula (Figure 1D asterisks; see also Additional file 2G). Taken together, in *ha* embryos, otolith mineralization is completely inhibited, even though major organic materials are supplied into the endolymph.

### *ha* gene encodes a polyketide synthase

Using positional cloning, we narrowed down the *ha* locus to a 64.7 kb region in linkage group 20, which contains four open reading frames (ORFs 1–4). Sequencing analysis identified a 9-nucleotide deletion (3-amino acid deletion) in ORF 2 that encodes a type I polyketide synthase (PKS) (Figure 2A). We thereafter named this gene *Oryzias latipes* polyketide synthase (*olpks*). The *olpks* transcript is 6153 nt in length, comprises six exons, and encodes a 2051 amino acid protein. PKSs are multifunctional enzymes mainly found in bacteria, fungi and plant, and catalyze the biosynthesis of a diverse group of second metabolite, polyketides, some of which are used for pharmaceuticals with antibiotic and mycotoxic properties [33]. Type I PKS has a set of distinct enzymatic domains that individually catalyze a series of reactions, to produce a final compound. Likewise, OlPKS contains five distinct domains: ketoacyl synthase (KS), acyl transferase (AT), dehydratase (DH), ketoreductase (KR), and acyl carrier protein domain (ACP) (Figure 2B). Essential amino acid sequence motifs for the function of each domain are conserved in OlPKS (Additional file 5B). The vertebrate fatty acid synthase (FAS), essential for all organisms, is thought to be an evolutionary subset of this family [34,35]. Animal FASs and another iterative type I PKSs share a conserved structure that includes KS, AT and ACP domains. FASs contain additional enzymatic domains such as KR, DH, ER and TE, which are present on different PKSs in different combinations. OlPKS possesses the minimal module (KS, AT and ACP) and additional KR and DH domains (Figure 2B and Additional file 5B).

We confirmed that *olpks* is indeed responsible for the *ha* phenotype by the following results, (i) phenocopy by injection of an antisense morpholino-oligonucleotide (MO) (Table 1), (ii) identification of a mutation of the *olpks* locus in another allele, *ki79* (Additional file 5A and B) which was isolated from a N-ethyl-N-nitrosourea (ENU)-driven screen (unpublished; screen conducted for medaka mutants with defects in bone or blood development at the Tokyo Institute of Technology, Japan), and (iii) phenotypic rescue by injection of full length *olpks* mRNA (Table 1). We also confirmed that all domains of OlPKS are indeed required for otolith formation by injecting mRNAs, each of which causes one amino acid substitution at one of the four enzymatic active sites and the essential site of ACP (Additional file 5B and Table 1).

**Table 1:** **Otolith formation in MO or mRNA of OlPKS**

**Experiment**	**Nucleltide**	**Fish**	**4 otoliths**	**1-3 otoliths**	**No otolith**	**n**
Phenocopy	*olpks* first-Met MO	*wt*	0%	0%	100%	39
Rescue	(uninjected)	*ha*	0%	0%	100%	31
	*olpks* mRNA		73%	23%	3%	30
Active site mutation	(uninjected)	*ki79*	0%	0%	100%	14
	*olpks*-mRNA		65%	35%	0%	43
	KS^*^-mRNA		0%	0%	100%	15
	AT^*^-mRNA		0%	2%	98%	57
	DH^*^-mRNA		0%	0%	100%	18
	KR^*^-mRNA		0%	0%	100%	17
	ACP^*^-mRNA		0%	0%	100%	27
	Loop^*^-mRNA		53%	40%	7%	60

Asterisks indicate one amino acid mutation are introduced.

‘Loop’: interdomain region.

Fully rescued: 4 otoliths in one animal.

Partially rescued: 1–3 otoliths in one animal.

Not rescued: no otolith.

The expression of *olpks* is transient and exclusively restricted to the OV in developing embryos. The expression initiates at the early somite stage (st. 19) and disappears between 16-somite (st. 24) and 19-somite stage (st. 25), a period when otolith mineralization initiates (Figure 2C and D). The expression becomes restricted to the medial and dorsal region of the vesicle at later stages (Figure 2C and Additional file 5C). The medaka genome has three *pks* related genes including *olpks* (Table 2) and we confirmed that the other two are not expressed at embryonic stages and adult tissues (Additional file 5D).

**Table 2:** **Type I PKSs found in animal lineage**

**Type 1 PKS?**	**Species name**	**General name**	**Gene name**	**Accession number**	**Reference genome**	**Gene ID**	**Corresponding RNA (Unigene or RNA)?**	**Domians**
	*Homo sapiens*	Human	-	-	Homo sapiens GRCh37.p13	-	-	-
	*Bos taurus*	Cattle	-	-	Bos taurus UMD3.	-	-	-
	*Canis (lupus) familiaris*	Dog	-	-	Canis lupus familiaris CanFam3.1	-	-	-
	*Tursiops truncatus*	Dolphin	-	-	Tursiops truncatus turTur1	-	-	-
	*Mus musculus*	Mouse	-	-	Mus musculus GRCm38.p1	-	-	-
✓	*Monodelphis domestica*	Opossum	PREDICTED: phthioceranic/hydroxyphthioceranic acid synthase-like	XP_001375980	MonDom5	LOC100024851	no	KS-AT-DH-KR-ACP
✓	*Sarcophilus harrisii*	Tasmanian devil	mycocerosic acid synthase-like [Sarcophilus harrisii (Tasmanian devil)]	XP_003771909	Devil_refv7.0	LOC100922065	N.A	KS-AT-DH-KR-ACP
	*Ornithorhynchus anatinus*	Platypus	LOC100091954 fatty acid synthase-like [Ornithorhynchus anatinus (platypus)]	-	Ornithorhynchus_anatinus-5.0.1	LOC100091954 (pseudo gene)	N.A	N.A (pseudo gene)
✓	*Gallus gallus*	Chicken	PREDICTED: phthioceranic/hydroxyphthioceranic acid synthase-like is oformX2 [Gallus gallus].	XP_418588	Gallus_gallus-4.0	LOC420486	Yes (brain; connective, blood)	KS-AT-DH-KR-ACP
✓	*Taeniopygia guttata*	Zebra finch	PREDICTED: phthioceranic/hydroxyphthioceranic acid synthase-like [Taeniopygia guttata].	XP_002189754	Taeniopygia_guttata-3.2.4	LOC100231542	No	KS-AT-DH-KR-ACP
			PREDICTED: Taeniopygia guttata phthioceranic/hydroxyphthioceranic acid synthase-like	XP_002190558		LOC100222288	N.A	KS-AT-DH-KR-ACP
✓	*Falco peregrinus*	Peregrine falcon	PREDICTED: probable polyketide synthase 1-like [Falco peregrinus].	XP_005234016	F_peregrinus_v1.0	LOC101916009	N.A	KS-AT-DH-KR-ACP
✓	*Anolis carolinensis*	Green anole	PREDICTED: phthioceranic/hydroxyphthioceranic acid synthase-like [Anolis carolinensis].	XP_003222100	AnoCar2.0	LOC100564455	No	KS*-AT-DH-KR-ACP
			PREDICTED: phthioceranic/hydroxyphthioceranic acid synthase-like [Anolis carolinensis].	XP_0032222101		LOC100564655	No	KS-AT-DH-KR-ACP
			PREDICTED: phthioceranic/hydroxyphthioceranic acid synthase-like [Anolis carolinensis].	XP_0032222102		LOC100564856	No	KS-AT-DH-KR-ACP
✓	*Chelonia mydas*	Green sea turtle	Phthioceranic/hydroxyphthioceranic acid synthase [Chelonia mydas].	EMP37033	CheMyd_v1.0	locus_tag: UY3_05838	N.A	KS-AT-DH-KR-ACP
			Polyketide synthase Pks N [Chelonia mydas]	EMP24664		locus_tag: UY3_18267	N.A	KS*-AT-DH-KR-ACP
✓	*Chrysemys picta*	Painted turtle	PREDICTED: uncharecterized protien LOC101936604 [Chrysemys picta bellii]	XP_005291085	Chrysemys_picta_bellii-3.0.1	LOC101936604	N.A	KS-AT*-DH*-KR-ACP
			PREDICTED: uncharecterized protien LOC101937174 [Chrysemys picta bellii]	XP_005291087		LOC1019371714	N.A	other-KS-AT*-DH*-KR-ACP
✓	*Pelodiscus sinensis*	Chinese soft shell turtle	pep: KNOWN_BY_PROJECTION_protein_coding	Scaffold no. JH209275.1	PelSin_1.0	ENSPSIG00000004874	No	KS*-AT-DH-KR-ACP
	*Xenopus (Silurana) tropicalis*	Tropical clawed toad	-	-	Xenopus (Silurana) tropicalis build 1 genome database (v4.2 assembly)	-	-	-
✓	*Oryzias latipes*	Medaka	OIPKS (PREDICTED: phthioceranic/hydroxyphthioceranic acid synthase-like [Oryzias latipes])	XP_004081385	Oryzias latipes ASM31367v1	LOC101169887	No	KS-AT-DH-KR-ACP
			OIPKS-2 (PREDICTED: phthioceranic/hydroxyphthioceranic acid synthase type I P ps D-like [Oryzias latipes])	XP_004081384		LOC101169644	No	KS-AT-DH*-KR-ACP
			OIPKS-3 (PREDICTED: probable polketide synthase 1-like [Oryzias latipes])	XP_004080917		LOC101170716	No	KS-AT-DH*-KR-ACP
✓	*Danio rerio*	Zebrafish	Danio rerio wu:fc01d11 (wu: fc01d11),mRNA	XP_682975	Danio rerio Zv9	wu:fc01d11	Yes (muscle)	KS-AT-DH-KR-ACP
			si: dkey-61p9.11	NP_001041530		LOC100000781	Yes (kidney)	KS-AT-DH-KR-ACP
✓	*Takifugu rubripes*	Fugu	phthioceranic/hydroxyphthioceranic acid synthase-like	XP_003968201	FUGU5	LOC101079294	No	KS-AT-DH-KR-ACP
			PREDICTED: Takifugu rubripes lovastatin nonaketide synthase-like (LOC101079519), mRNA	XP_003968202		LOC101079519	No	KS-AT-DH-KR-ACP
	*Petromyzon marinus*	Lamprey	-	-	Pmarinus_7.0	-	-	-
✓	*Branchiostoma Floridae*	*Lancelet*	hypothetical protien BRAFLDRAFT_205831, partial [Branchiostoma floridae].	XP002599684 (=EEN55696)	Branchiostoma floridae v1.0	Gene ID:7231796	N.A.	KS-AT-DH-KR
			hypothetical protien BRAFLDRAFT_247081 [Branchiostoma floridae]	XP_002591573		Gene ID:7219804	N.A.	KS-AT-DH*-KR
			hypothetical protien BRAFLDRAFT_90481 [Branchiostoma floridae]	XP_002589799		Gene ID:7210376	N.A.	KS-AT-DH*-KR-AMP
			hypothetical protien BRAFLDRAFT_96868 [Branchiostoma floridae]	XP_002598386		Gene ID:7254845	N.A.	KS-AT-DH-KR*-TE-C-AMP
			hypothetical protien BRAFLDRAFT_96863 [Branchiostoma floridae]	XP_002598380		Gene ID:7248951	N.A.	KS-AT-DH-KR*-ACP-TE-C-AMP
			hypothetical protien BRAFLDRAFT_87472 [Branchiostoma floridae]	XP_002589000		Gene ID:7246004	N.A.	KS-AT-DH-KR*-ACP-C-AMP
			hypothetical protien BRAFLDRAFT_125690 [Branchiostoma floridae]	XP_002610053		Gene ID:7207083	N.A.	KS*-AT-DH-KR*-ACP*-TE-C
			hypothetical protien BRAFLDRAFT_91451 [Branchiostoma floridae]	XP_002608071		Gene ID:7214024	N.A.	KS-AT-DH-MT-ADH-KR-ACP-α
			hypothetical protien BRAFLDRAFT_87410 [Branchiostoma floridae]	XP_002605916		Gene ID:7243248	N.A.	KS-AT-DH-MT-ADH-KR-ACP
			hypothetical protien BRAFLDRAFT_89867 [Branchiostoma floridae]	XP_002610100		Gene ID:7206066	N.A.	KS-AT-DH-MT-ADH-KR-ACP
			hypothetical protien BRAFLDRAFT_87413 [Branchiostoma floridae]	XP_002605913		Gene ID:7246000	N.A.	KS-AT-DH-MT-ADH-KR-ACP
			hypothetical protien BRAFLDRAFT_125650 [Branchiostoma floridae]	XP_002610103		Gene ID: 7207596	N.A.	AMP-KS-AT-DH-MT-ADH-KR-ACP
			hypothetical protien BRAFLDRAFT_71890 [Branchiostoma floridae]	XP_002613500		Gene ID: 7224978	N.A.	KS*-AT-DH
✓	*Saccoglossus kowalevskii*	Acorn worm	PREDICTED: fatty acids synthase-like	XP_002734101	Skow_1.1	LOC100373061	N.A	KS*-AT-DH-KR-ACP-C-AMP
	*Ciona intestinalis*	Ascidian	-	-	Ciona intestinalis KH	-	-	-
✓	*Strongy locentrotus purpuratus*	Purple sea urchin	LOC588806 probable polyketide synthase 1-like [Strongylocentrotus purpuratus (purple sea urchin)]	XP_793564.2	Spur_3.1	LOC58806	Yes	KS-AT*-DH*-MT-ADH-KR*-ACP
			LOC592147 polyketide synthase 2	NP_001239013.1		LOC592147	Yes	KS-AT-DH*-KR-ACP-TE
✓	*Acropora digitifera*	Coral	*aug_v2a.12941.t1 aug_v2a.12941scaf5202:2805-26950(−)*	aug_v2a.12941	Adig_1.0	aug_v2a.12941	N.A.	other-KS-AT-DH-KR-ACP*-TE
			aug_v2a.16843.t1 aug_v2a.16843scaf8086:11276-29700(−)	aug_v2a.16843		aug_v2a.16843	N.A.	other-KS-AT-DH-KR-TE
			aug_v2a.16847.t1 aug_v2a.16847scaf8086:95926-115499(−)	aug_v2a.16847		aug_v2a.16847	N.A.	other-KS-AT-DH-KR-TE
	*Nematostella vectensis*	Sea anemone	-	-	Nematostella vectensis v1.0	-	-	-
	*Hydra magnipapillata*	Hydra	-	-	Hydra magnipapillataHydra_RP_1.0	-	-	-
	*Amphimedon queenslandica*	Sponges	-	-	Amphimedon queenslandicav1.0	-	-	-
✓	*Caenorhabditis elegans*	Nematode	Protein C41A3.1 [Caenorhabditiselegans]	NP_508923	Caenorhabditis elegansWBcel235	C41A3.1	Yes	KS-KS-DH-ACP-KS-ACP-ACP-KR-ACP-AT-DH-KS-KR-KS-AT-ADH-ACP-C-AMP-ACP-TE
	*Drosophila melanogaster*	Fruit fly	-	-	Drosophila melanogasterRelease 5	-	-	-

Asterisks show domains lacking a residue of the active site that is contained in OlPKS.

Sequence sources are mainly NCBI protein database, except dolphin, Chinese soft shell turtle, lamprey, coral, and sea anemone.

KS: ketoacyl synthase, AT: acyl transferase, DH: dehydratase, KR: ketoreductase, ACP: acyl carrier protein domain, TE: thioesterase, AMP: AMP-binding site, MT: methyltransferase, ADH: alcohol dehydrogenase, C: condensation domain.

Based on above results, we conclude that OlPKS is only required for the early step of otolith mineralization and that the mutation in *olpks* is responsible for the *ha* phenotype.

### OlPKS produces lipophilic substances secreted into the endolymph

We hypothesized that, similar to other PKSs, OlPKS synthesizes polyketide-related small compounds in the OV epithelium, which are then secreted into the endolymph for the initial step of otolith mineralization.

**Figure 3:**
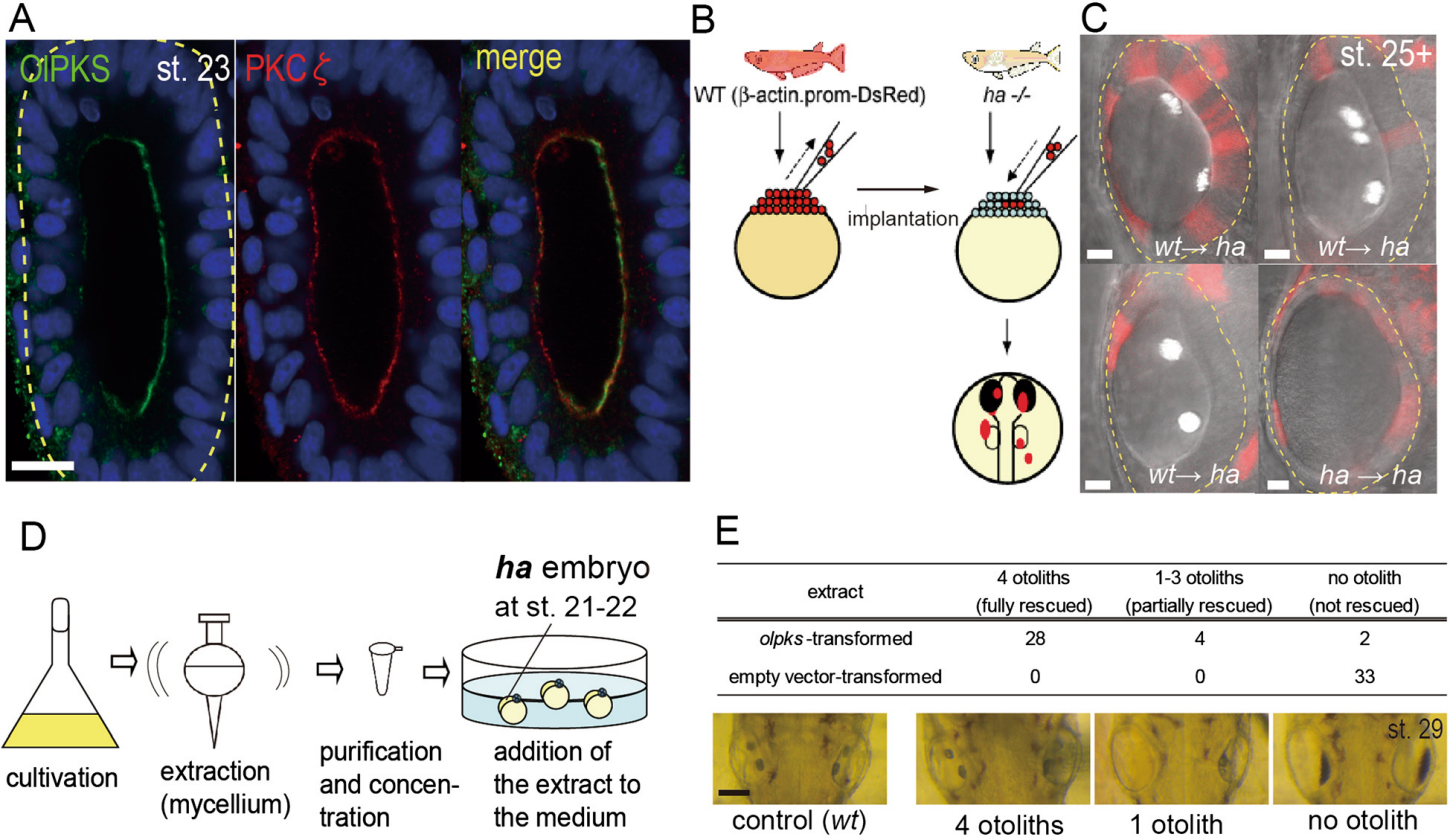
**OlPKS produces lipophilic substances secreted into the endolymph. (A)** Intracellular localization of OlPKS in the OV epithelium (dorsal views of the left OV). Anti-OlPKS antibody detects the OlPKS protein at the apical region of the epithelial cells. Co-immunostaining with a membrane marker, PKC ζ, shows it localizes near the apical membrane. Yellow dotted line: OV. Scale bar: 10 μm. **(B)** Schematic procedure of the chimeric experiment. **(C)** Some images of the chimeric OVs in live embryos. [*wt* → *ha*] shows *wt* cells expressing DsRed are transplanted to an *ha* embryo. [*ha* → *ha*] is a negative control experiment. Yellow dotted lines: OVs. Scale bars: 10 μm. **(D)** Schematic representation of the heterologous expression system using *A. oryzae.* **(E)** Summary of the bioassay in the heterologous expression experiment. Numbers of *ha* embryos treated by the extract of *olpks* transfromant or that of empty vector toransformant are shown (*Upper table*). Grades of recovery of the mineralization: four otoliths (two per OV, fully rescued), 1–3 otoliths (at least one otolith per embryo, partially rescued) and no otolith (not rescued). Representative picture of treated embryo in each category is shown with a picture of *wt* embryo (*Lower*). Scale bar: 100 μm.

To test this idea, we first examined subcellular localization of OlPKS. Immunostaining revealed that the medial wall of the OV exclusively expresses OlPKS at st. 23, which is highly localized at the apical of epithelial cells (Figure 3A *Left*) (a region for antigen is described in Additional file 5B). Double staining with an antibody to PKC ζ (Figure 3A *Center*), an apical membrane marker, demonstrated that the distribution of OlPKS enzyme partially overlaps with that of PKC ζ but OlPKS signal is detected closer to the lumen (Figure 3A *Right*). Thus, substances synthesized at the apical surface can be directly secreted to the endolymph, in a way similar to ‘membrane-localized’ PKS observed in bacterial cells [36,37].

Next, we performed a chimera experiment in which DsRed-expressing *wt* cells was transplanted into mutant blastula, and examined for otolith formation when *wt* donor cells colonized mutant OVs (Figure 3B). Remarkably, irrespective of their number and localization within the OV, *wt* cells effectively restored otolith formation at the appropriate time and location, the macula region, in *ha* embryos. Only a few cells, located at any region of the vesicle, were found to be sufficient (Figure 3C and Additional file 6A and B).

Finally, we adopted a heterologous expression system to characterize substances synthesized by OlPKS. Since large-scale expression of PKSs has been established in *Aspergillus oryzae* [24], we introduced the *olpks* cDNA into *A. oryzae*, expecting that exogenous PKS (i.e., OlPKS) could work using endogenous substrates such as acetyl-CoA and malonyl-CoA in fungal cells like their own PKSs (Figure 3D). OlPKS expression in transformed fungi was confirmed by western-blotting (Additional file 6C). Since polyketide-derivatives exhibit moderate hydrophobic nature, we extracted cultivated mycelia with acetone, followed by purification using partition between ethyl acetate/H_2_O and subsequent purification. We then attempted a simple *in vivo* assay in which *ha* mutant embryos were cultured with an aliquot of the extracts (Figure 3D). Remarkably, these extracts restored otolith mineralization in *ha* embryos, while no such rescue was observed with control extracts (Figure 3E). Taken together, these data demonstrate that it is not OlPKS but ethyl-acetate extractable substances synthesized by OlPKS and secreted into the endolymph that nucleate otolith mineralization.

### Broad distribution and conserved roles of polyketide synthases in animals

**Figure 4:**
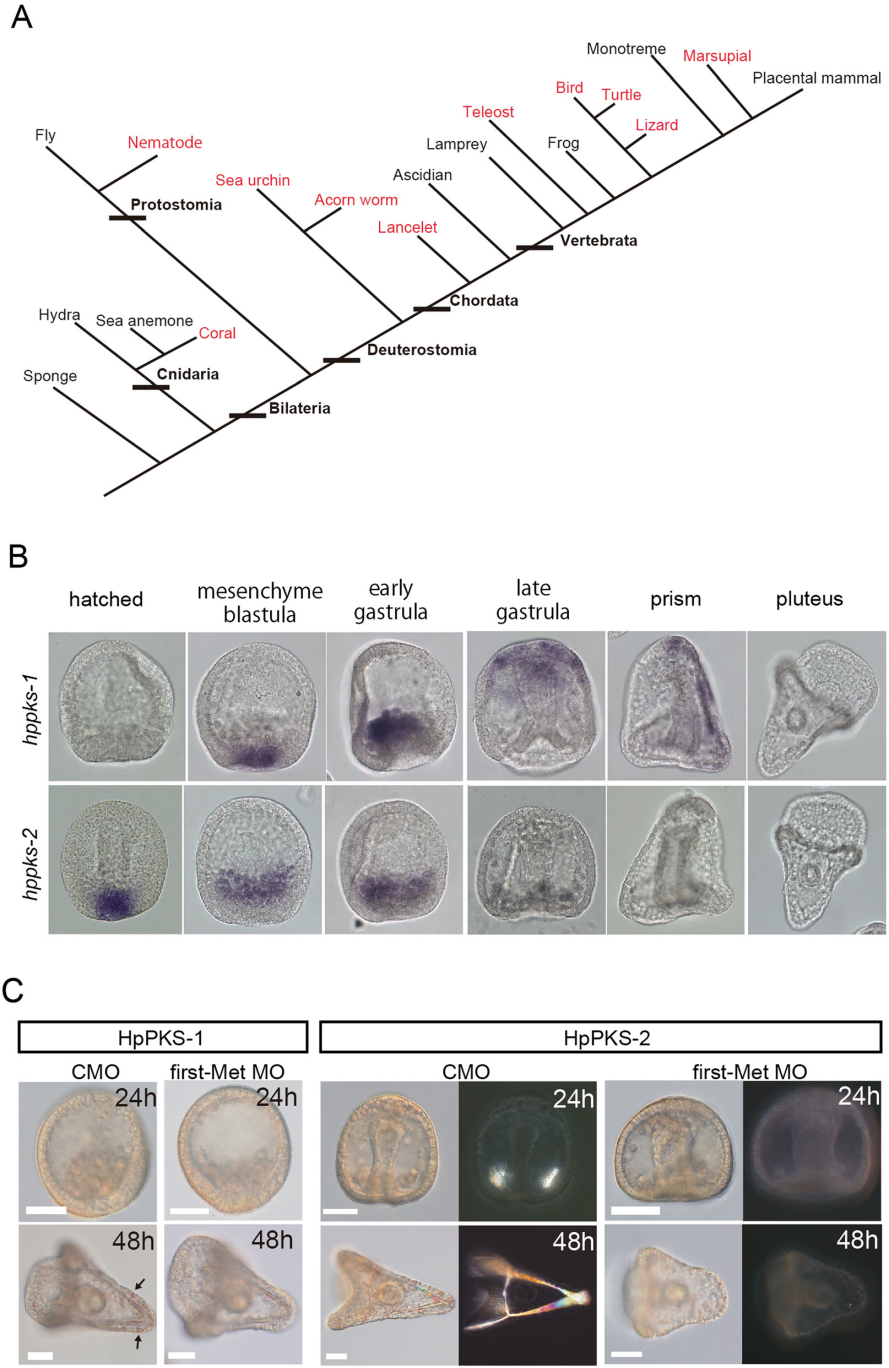
**Broad distribution and conserved roles of PKSs in animals. (A)** Distribution of the *pks* genes found by the BLAST searches in the schematic phylogenetic tree of animal kingdom. Red font shows the presence of type I *pks* gene(s) in the species. Except for fly, frog and mammal, most intensively studied models, *pks* genes could be overlooked due to incomplete genome information. **(B)** Whole-mount *in situ* hybridization of *H. pulcherrimus* with probes for *hppks-1* (*Upper Panel*) and *hppks-2* (*Lower Panel*). *hppks-1* was first detected at the mesenchyme blastula stage in the precursors of the secondary mesenchyme cells (SMCs) at the vegetal pole, and the expression persisted until the prism stage, in the SMCs and then in the ectoderm. The expression was no longer observed in pluteus larvae. *hppks-2* expression initiates in PMC precursors at the blastula stage and disappear by late gastrula just after spicule formation starting (mid-gastrulation). **(C)** Representative results of the MO knockdown experiments in *H. pulcherrimus*. Images were taken at two stages (24 h and 48 h). Arrows indicate pigment cells. HpPKS-2 first Met MO-injected or its control MO-injected embryos were also observed by a dark-field microscope for visualizing the spicules. Each MO was injected at a concentration of 200 μM. ‘CMO’: Control MO, Scale bars: 50 μm.

Animal *pks* genes were rarely explored, except for two echinoderm *pks-1* and *pks-2* (isolated from *Strongylocentrotus purpuratus*) [38,39], and the presence of fish, bird, and nematode *pks* genes were reported by previous phylogenetic analyses [40-42]. We performed thorough database searches for animal *pks* genes against current updated genome databases. Identified *pks* gene candidates were then assessed for a constitution of domains in each predicted amino acid sequence. These searches revealed a remarkably broad distribution of *pks* genes from Cnidaria to Bilateria, including coral (*Acropora digitifera*), *C. elelgans* and reptiles/birds (Figure 4A and Table 2). Usually, 1–3 *pks* genes are present in each genome, except for the lancelet genome that contains 13 genes. Most vertebrate PKSs have five domains, which are similar to OlPKS (*e.g.*, KS, AT, DH, KR and ACP; Table 2). Zebrafish *pks*, *drpks* (*wu:fc01d11*), expresses in the otic vesicle (Additional file 7B). By contrast, other animal PKSs are not similar to *olpks* and have versatile domains, especially *C. elegans* PKS contains 21 domains in the polypeptide (predicted by Pfam search) [42]. The phylogenetic tree constructed using the sequence of KS domain (Additional file 7A) shows that animal PKSs are phylogenetically distinct from animal FASs and rather they are close to microbial PKS genes (Additional file 7A).

We hypothesized that like medaka PKSs, some of the other animal PKSs participate in biomineralization, more specifically calcium carbonate mineralization. To test this idea, we focused on echinoderm *pks-2* because its reported expression is in primary mesenchyme cells (PMCs) that give rise to spicules, larval skeletons made of calcium carbonate [38,43]. We first confirmed the PMC-specific expression of *pks-2* in our experimental system, *Hemicentrotus pulcherrimus* (*hppks-2*). Importantly, the expression of sea urchin *pks-2* disappears around late gastrula stage just after PMCs begin to form spicules (Figure 4B *Lower Panel*). We then examined the function of HpPKS-2 by injecting HpPKS-2 MO. As shown in Figure 4C, HpPKS-2 morphants exhibited severe defects in spicule formation while control MO-injected embryos appeared normal (Figure 4C and Additional file 7C). Another echinoderm *pks* gene, *hppks-1* was found to contribute to pigmentation as previously reported [39] (Figure 4B *Upper Panel* and C, and Additional file 7C). We thus conclude that echinoderm *pks-2* plays a critical role in the formation of calcareous skeletal elements in larva.

## Discussion

We here show that *olpks* encoded by the *ha* locus is essential for the early step of otolith biomineralization in medaka. In *ha* mutant embryos, mineralized stones never form, but instead OMP-1-positive particles precipitate in the endolymph. Our TEM observation confirmed that crystal growth is never initiated in *ha* OV despite the accumulation of otolith materials (Figure 1D), suggesting that the product of OlPKS participates in the initial step, in particular, nucleation of mineralization. The expression pattern of *olpks* supports its early function; the expression is transient and disappears by the onset of otolith formation (Figure 2D). This pattern of expression contrasts sharply with that of other essential genes identified so far. For instance, *otolin-1* and *omp-1* expression begins at st. 25 and persists into adulthood as these proteins continue to be deposited on growing otoliths throughout life [44,45]. Furthermore, the expression of medaka *starmaker* (called *starmaker-like*), a key regulator of the crystal lattice of calcium carbonates [46], is detected at st. 21 and is again maintained at later stages [47]. Intriguingly, *ha* fry develop an otolith-like stone (Additional file 2H), and some adult *ha* fish finally have otolith-like stones, albeit with abnormal shape and size [48]. This does not occur in zebrafish ‘no-otolith’-type mutants such as *backstroke* and *keinstein* [49]. These facts further suggest that the endolymph of *ha* only lacks a trigger. In *ha*, continuous supply of otolith materials without crystallization could lead to abnormal development of the inner ear and irregular stone formation at later stages. Together with these phenotypic and expression analyses, we conclude that the product of OlPKS functions as a nucleation trigger (facilitator).

Despite our efforts to identify the product of OlPKS, it remains elusive; we failed to detect any specific peak of the synthesized compound in HPLC analysis using extracts of *A. oryzae* transformed with *olpks*, probably due to low production of the compound. However, the active substance could have amphiphilic nature like other polyketide (*e.g.*, phenolic lipids [50]) because it was re-solved in medaka culture medium and penetrates into the otic vesicle of the embryo in the rescue experiment (Figure 3D). Given its amphiphilic nature, the product of OlPKS could serve as a bridge between ACC and organic components such as soluble matrix protein (*e.g.*, OMP-1) and/or insoluble scaffold matrices (*e.g.*, Otolin-1)(the possible functions of these organic compounds in biomineralization were proposed by Nagasawa [3]). Supporting this idea, in studies of High-resolution Cryo-TEM imaging, monolayers of fatty acids (arachidic acid or stearic acid) that are also amphiphilic in nature, have been used to artificially induce crystal nucleation [7,51]. Further investigation using cell-free systems [52,53] will be necessary to address at which step of biomineralization PKS products actually work.

Otolith formation shares many features with the formation of vertebrate bones consisting of calcium phosphate (hydroxyapatite) minerals, the best characterized calcification process to date [54]. Intriguingly, in bone formation, phospholipid such as phosphatidylserine (PS) has been implicated in an inducer of hydroxyapatite mineralization and most likely acts as a nucleation facilitator [52,55,56]. Since PS is a small substance with amphiphilic nature, the product of OlPKS could be a functional counterpart in calcium carbonate mineralization, interacting with calcium ions, ACC and matrix proteins.

The phylogenetic analysis based on the KS domain demonstrates the topology in which animal PKSs are separated from the animal FAS clade (Additional file 7A). Unlike the *fas* gene, the distribution of *pks* genes in animals is irregular; they are not found in some animal groups, for examples, fly, frog, and mammal, which represent some of the most intensively studied model animals (Figure 4A). These facts complicate the evolutionary origin of animal *pks* genes, while a single origin of animal *fas* has been repeatedly supported. Horizontal gene transfer, gene duplications and gene losses could have occurred during evolution, as proposed in bacteria/fungi PKSs [57-59]. Among those animal *pks* genes, sea urchin *pks-2* could be functionally equivalent to *olpks* because of its early and transient expression in the calcifying cell lineage and its knockdown phenotype, although the domain architecture of sea urchin PKS-2 differs to some degree from that of OlPKS, being KS-AT-DH-ER-KR-ACP-TE and KS-AT-DH-KR-ACP, respectively [38].

Given the conserved role of medaka and sea urchin PKSs, calcium carbonate biomineralization could generally require the products of PKSs for nucleation. PKSs could participate in the production of coral skeletons and calcification of algae in the ocean. For instance, *Emiliania huxleyi* [58,60] has some *pks* genes (*e.g.*, *fgeneshEH_pg.50__74*, protein ID103465, Joint Genome Institute (http://genome.jgi-psf.org/pages/search-for-genes.jsf?organism=Emihu1)). Intriguingly, the presence of PKS in Cnidaria appears to be associated with biomineraliztion; coral (*A. digitifera*) has a couple of *pks* genes, whereas *Hydra magnipapillata* and *Nematostella vectensis* do not. Furthermore, *pks* genes of bird and reptile might be involved in production of egg shells. In frogs and mammals, however, *pks* genes were no longer needed and lost during evolution, although their inner ears still contain calcium carbonate biominerals. This inconsistency may be explained by the fact that their biominerals do not form large stones, but instead small grains called otoconia. However, further intense search in those animals which appear to have lost *pks* genes will definitely be needed.

## Conclusions

The present study addresses the function of the vertebrate *pks* gene and demonstrates its vital role in calcium carbonate biomineralization. Further functional analyses of newly identified PKSs will uncover a long-overlooked world of polyketides and their derivatives in animals. Our finding also provides genetic and molecular cues for the geochemical study of global carbon and calcium cycles.

### Accession numbers

*pks* genes reported herein have been deposited in GenBank with accession numbers as follow: *olpks* [AB923905], *hppks-1* [AB923906] and *hppks-2* [AB923907].

## Additional files

Additional file 1:
**Materials and Methods.**
Additional file 2:
**Phenotypic analyses in** ***ha mutant.*** (A-F) Whole-mount in situ hybridization of molecular markers expressed in the OVs. (A)(B) Differentiation markers, *eya-1* (across the vesicles) and *pax-2* (medial region) are normally expressed in *ha* embryos. All OVs are dorsal views. (C)(D) Normal expression patterns of marker genes. *dlx-3b* is restricted to the dorsal wall of the OV and *bmp-4* marks the neural cristae regions. ac,anterior cristae; lc, lateral cristae; pc, posterior cristae. All OVs are lateral views. Left side shows anterior. (E)(F) The transcripts of otolith matrix proteins, *starmaker-like* and *sparc-1* (are comparably detected in the mutant OV. All OVs are dorsal views. (G) TEM images from a *wt* specimen and a mutant one (representative images are shown in G to G’’’). Asterisks: growing otolith precursors; Arrows: fine substances; ‘s’: seeding particles; ‘g’: globules. (H) Immunohistochemistry for OMP-1 revealed crystalized ‘otolith’ of *ha* in hatching stage (st. 40) that looks similar to *wt* otolith and contains at least some organic materials. (I) double colored-Immunofluorescence of acetylated *α*-tubulin and γ-tubulin (root of cilia). Short cilia (<1μm) are visible in the OV of *ha* as well as *wt*. (J) Scatter plot showing total number of cilia in the OV of *wt* and ha at various stages. Total number of cilia in the OV is obtained by counting the signal of *α*-tubulin and *γ*-butulin. In each stage, there was no significant difference in total number of cilia between *wt* and *ha* (>0.05 Student’s t-test). (K) Otolith development in the OV of *ktu* mutant. In *ktu*, seeding particles are coalesced at the prospective macula region (*Upper*; dorsal views of left OV), and otoliths appear normal at later stage just like *wt* (*Middle*; dorsal views of left OV and *Lower*; lateral views of left OV). Asterisks: otoliths, Arrow: paste-like substance. Additional file 3:
**Movement of seeding particles in** ***wt*** **OV at st 25.** The movie is recorded at the rate of 1 frame per second and played back 5 times faster than real-time viewing (dorsal view of left OV). A number of seeding particles are floating and otoliths are already mineralized. Additional file 4:
**Movement of seeding particles in** ***ha*** **OV at st 25.** The movie is recorded at the rate of 1 frame per second and played back 5 times faster than real-time viewing (dorsal view of left OV). *ha* OV contains large number of seeding particles due to failure of mineralization. Additional file 5:
**Mapping analyses and expression pattern of the** ***ha*** **gene.** (A) Sequencing of the *ha* gene in two mutant fish. *ki79* fish harbors a nonsense mutation at 3856 nt of the ORF2 locus. (B) Architecture of OlPKS protein predicted from the amino acid sequence (*Upper*). Critical residues of each domain used for the mRNA injection experiment are shown by arrows. Mutation site of each mutant is shown by arrowhead. Three amino acids corresponding to 9-bp deletion is ‘KPS’. Central proline is found to be a highly conserved residue among PKSs although it is not previously considered as a conserved motif. Based on the structure of mammal fatty acid synthase (FAS), it is possible that the proline contribute to help a substrate to enter the active site of KS. The red bar indicates the region used as an antigen for producing anti-serum of OlPKS. Conserved motifs found in the amino acid sequence of OlPKS (*Lower table*). Asterisks show residues mutated in mRNA rescue experiments. Underlines show conserved amino acid residues. (C) Whole-mount *in situ* hybridization with *olpks* probes at st. 22. The dorsally- and medially- restricted pattern is evidenced by lateral view (*Upper*) and histology of the OV region (*Lower*). Yellow dashed lines show OVs. (D) Expression profiles of *olpks* and paralogous gene candidates in the embryonic stages or adult tissues. A RT-PCR analysis reveals that *olpks* is expressed but *olpks-2* and *olpks-3* are not expressed and that *olpks* is expressed only around embryonic stage 22. Additional file 6:
**Analyses of property of the OlPKS product.** (A)(B) Imaging analyses of the chimeric experiment. (A) All 110 OVs of 55 transplanted animals are observed and sorted to 9 categories depending on their percentage of *wt* cells in their OV epithelium (Note a chimeric fish has two OVs). The histogram shows that a very small number of *wt* cells can rescue otolith formation. (B) The same sample set is also analyzed to assess the localization of *wt* cells within the OV. ‘medial’ means *wt* cells located around medial wall of the OV region where *olpks* gene is normally expressed in *wt* animal; ‘medial and other’ includes medial and any other epithelial region; ‘other’ includes *wt* cells existing anywhere in the epithelium except the medial wall. (C) Western blot of OlPKS protein produced by *A. oryzae*. To verify expression of OlPKS in heterologous expression experiment, purified protein from a transformant is analyzed by anti-OlPKS antiserum. The OlPKS protein gave a protein band at a position of ca. 210 kDa on SDS-PAGE. However, some bands (degradade proteins) were also found in this experiment. Additional file 7:
**Broad distribution of animal PKSs.** (A) Maximum parsimony phylogenetic estimate of relationships among animal type I PKSs and FASs, based on an alignment of amino acid sequences of the KS domain. Animal PKSs containing OlPKS are not divided from other bacterial or fungal PKSs, as indicated by low values of the bootstrap. By contrast, the animal FAS clade is clearly separated from animal PKSs. Protein accession number of Genbank or other database is shown with species name. Numbers described around branches indicate percentage of the bootstrap value supporting each clade. Branch length indicates number of inferred amino acid changes. (B) Representative image of whole-mount *in situ* hybridization of zebrafish embryo using probes for *pks* homologue. The transcript of a *pks* homologue, *drpks(wu:fc01d11)*, is exclusively expressed in zebrafish OV at the 20 somite-stage (19hpf). Arrows: OVs. (C) Summary of knockdown experiment using MOs. Injected embryos are categorized by the phenotype of spicule. HpPKS-1 knockdown animal lacked pigment while HpPKS-1 control MO (CMO)-injected animals showed normal pigmentation. These two morphants didn’t show any spicule abnormality. Injection of HpPKS-1 MO and HpPKS-1 CMO at high concentration (200 μM) caused developmental arrest at the gastrulation. Various concentrations of HpPKS-2 MO injection (three levels; 50, 100 and 200 μM) shows dose dependency. All HpPKS-2 CMO-injected embryos showed no spicule defect. Standard control MO did not give any effect.
