# Supplementary material for: Unexpected link between polyketide synthase and calcium carbonate biomineralization
Source: Zoological Lett. 2015 Jan 13;1:3. doi: 10.1186/s40851-014-0001-0 (PMC4604110; doi:10.1186/s40851-014-0001-0)
Supplement: Additional file 1: — Materials and Methods. [file 40851_2014_1_MOESM1_ESM.docx]

**Additional file 7**

**Materials and Methods**

**Whole-mount *in situ* hybridization for medaka embryos.** Whole-mount *in situ* hybridization analyses were performed as previously described [[1](#_ENREF_1)]. For histological analysis, whole-mount *in situ* hybridization samples were embedded in Technovit 8100 resin (Heraues-Kulzer) and sectioned into 5-7 μm-thick sections. cDNAs used as the templates for probe were isolated from the d-rR strain by RT-PCR, except *pax2* (provided by Dr, Wittblodt, Germany [[2](#_ENREF_2)]). The partial fragments of *eya-1*, *dlx3b*, *bmp4*, *stm-l* and *sparc* were isolated by using following specific primers (5’to 3’): *eya-1*, CAGGAGAGTACAGTACCATCCACA and CCTGTCCGAGCCCTGGCGGCTGCA [[3](#_ENREF_3)]; *dlx3b*, GGGATCCATGAGCGCCGGACAGACC　and GCTCGAGATAAACAGCTCCCACGCTCT [[3](#_ENREF_3)]; *bmp4*, TGCTCTATTTTCTGTCGAGACATC and GTTGTGTGGAGTCTGATTAAGGTG; *starmaker-like*

, GCGAGGAATTGGATTCAGCAGATGA and GCCTGGCAAGGCAAACAAACAGGAA [[4](#_ENREF_4)] ; *sparc*, AGGGGGAGTTTGAGCACATC and AGGGATCAGCACCATTTCTG [[5](#_ENREF_5)]. For the expression analyses of *olpks*, cDNAs used as the templates for probe were isolated by RT-PCR from the d-rR strain at st. 22. The full length of *olpks* was isolated by using following specific primers: ATGGAGGACGGAATAGCCGTGG and TTAGCTGCTGCTGTGGACAAACTCC.

**Observation of seeding particles**

Embryos were dechorionized and mounted in 2% low-melting point agarose in a grass-bottom petri dish. Seeding particles was observed at the rate of 1 frame per second using a confocal microscopy (Zeiss, LSM 710).

**RT-PCR.** RT-PCR analysis of *olpks* was performed using total RNA from embryos, fry and adult tissues. cDNA libraries were generated using the SuperScript First-Strand Synthesis System III for RT-PCR (Invitrogen). The primers 5’-CAAGTGTTGCAGCCAATAGAATTTC-3’ and 5’-CTCCTGCCACCCTCCCGTGAGGCCCT-3’ were used to amplify a 780 bp fragment of *olpks*. In addition, following primers were used to amplify expected 896bp and 1047bp fragments of *olpks-2* (LOC101169644: XP_004081384) and *olpks-3* (LOC101170716: XP_004080917), respectively (5’to 3’): *olpks-2*, GAAACAAACGCCGCACACATGCATC and CTTTGCTGGCATCTCATCGCTCTTT; *olpks-3*, CAGATTTGACTTAGCGGGCTGGTAT and GAACTGAGAGATCAAAGACGTCACCT.

**Immunofluorescence.** Immunofluorescence for OMP-1 was performed as previously described by Murayama *et al*. [[6](#_ENREF_6)], whereas staining for OlPKS and PKC ζ were done as previously described by Kamura *et al.* [[7](#_ENREF_7)]. For OMP-1 staining, embryos were fixed with 4% paraformaldehyde/PBS (phosphate buffered saline) overnight at 4C. After washing with PBST (0.1% TritonX-100 in PBS), the samples were dehydrated with methanol and stored at -20C. The samples were rehydrated with PBST, then treated with the blocking solution (5% sheep serum, 0.2% BSA in PBST) for 2 hours at room temperature. Primary and secondary antibodies were diluted in blocking solution. As primary antibody, anti-rainbow trout (*Oncorhynchus mykiss*) OMP-1 serum was used at a 1:500 dilution [[6](#_ENREF_6)]. The embryos were incubated with the serum overnight at 4C. After washing with PBST for at least 2 hours, the embryos were blocked again then incubated overnight with Alexa 488 (Molecular Probes) conjugated secondary antibodies at a 1:400 dilution. For OlPKS and PKC ζ staining, embryos were fixed with methanol/DMSO mixture (methanol:DMSO = 4:1) 15 minutes at 4C, blocked in 2% BSA/PBSDT (10% DMSO/0.2% Triton X-100/PBS) and incubated with primary antibodies in 2% BSA/PBSDT overnight at 4C: anti-OlPKS serum at a 1:50 dilution; PKC ζ (Santa Cruz, sc216-G) at a 1:50 dilution. After washing in PBSDT, embryos were incubated with Alexa 488 or Alexa 555 or Cy5 (Chemicon) conjugated secondary antibodies at a 1:400 dilution. Washed embryos were transferred in 50% glycerol/PBS and photographed using a LSM710 confocal fluorescence microscope (Zeiss). Polyclonal rabbit OlPKS [amino acids 1520-1711] antibodies were raised by immunization of rabbits with bacterially expressed His-tagged truncated proteins.

**Immunohistochemistry.** For histological analysis, larvae were fixed 4% PFA/PBST for 3 hours and incubated in 10% EDTA/PBS for 2 days to decalcify. Specimens were embedded in Technovit 8100 (Heraeus Kulzer) and cut into 6μm thick sections. Sections were treated with 1.5% H_2_O_2_ to block endogenous peroxidase. After sections were washed in PBST, they were blocked with 2% goat serum diluted in PBS. Primary antibody (OmOMP-1) was diluted in PBS (1:8000) and incubated for overnight at 4C. After incubation, sections were washed and incubated with secondary antibody (anti-rabbit IgG-Biotin; SIGMA, B7389) overnight at 4C. After washing, sections were incubated with an ABC Kit solution (Vector, #PK6101) for 15 minutes at room temperature. Finally, they were rinsed in PBST and incubated in DAB (0.2 mg/ml PBS; 0.03% H_2_O_2_) for 15 minutes. To stop the reaction, sections were rinsed in DW, after which they were dried and mounted by Entellan® new (Merck Millipore).

**Genomic research.** Animal type I PKS sequences were retrieved by TBLASTN against genome sequences of each species or BLASTP search against nr-protein database using the OlPKS sequence. TBLASTN parameters: default settings at NCBI TBLASTN, except [Word size = 2] and [No Filter against low complexity regions]. For each candidate, domain search was done by Pfam sequence search, and then assessed for the presence of an animal-FAS homologous sequence and the constitution of domains; the basic domains for type I PKS, KS and AT, as well as additional domains (i.e., DH, KR, ACP etc.).

**Phylogenetic analysis.** To draw the molecular phylogenetic tree, we used three groups of *pks* related genes; Animal type I PKSs (listed in Table 2), animal FASs and non-animal PKSs. We searched for animal FASs using BLASTP against the genome database of major model animals and some species that have non-FAS PKS. The complete amino acid sequence of human FAS (NP_004095) was used as the query to retrieve animal FASs. Additionally, we thoroughly searched each group of non-animal organisms (e.g., plants, fungi and bacteria) for sequences similar to OlPKS (full length amino acid sequence) by conducting individual BLASTP searches against each group independently. Some type II PKSs were used as outgroups. Multiple sequence alignment was performed using CLUSTAL W and the Maximum parsimony phylogenetic tree was constructed using the RAxML web service [[8](#_ENREF_8)] and NJ plot program. As previously reported [[9](#_ENREF_9), [10](#_ENREF_10)], we conducted phylogenetic analyses using KS domain (approx. 400 amino acid length), the most conserved domain of typeI PKS.

**Whole mount *in situ* hybridization for zebrafish embryos.** zebrafish (TuAB) embryos were fixed around 19 hpf. Whole-mount *in situ* hybridization was basically performed as previously described [[1](#_ENREF_1)], except blocking solution for anti-DIG antibody (3% sheep serum/PBST). cDNAs used as the templates for probe of *drpks* (*Danio rerio* PKS gene), *wu:fc01d11* (XP_682975), were isolated from the TuAB strain by RT-PCR. The partial fragment of *wu:fc01d11* was isolated by using following specific primers: 5’-GGAGTAAGAATTCTTACTCTTCAGT -3’and 5’-GTCCTCATTGGTGCTCTGTTTTAA-3’.

**Generation the *ha* chimeric medaka.** Transplantation experiments were performed according to previous work [[11](#_ENREF_11)]. [Tg (β-actin:DsRed)] were used as donors, whereas *ha* fish were used as hosts. At the morula stage, both the donors and hosts were dechorionated by hatching liquid. At the mid-blastula stage, the embryos were placed on V-shaped grooves of a 1.5% agarose gel immersed in Yamamoto’ s Ringer solution (the Ringer's solution for medaka), and then the cells were transplanted to the prospective head area of the embryo, using a micromanipulator (Narishige, M-152) in combination with a microinjector (Narishige, IM-6). After incubating these embryos overnight, we discarded the embryos whose ear region is largely occupied by *wt* cells. For the control experiment, *ha* fish ubiquitously expressing DsRed were obtained: [Tg (β -actin:DsRed)] and *ha* medaka were mated, and F_1_ fish expressing DsRed were crossed with each other, followed by F_2_ screening for *ha* phenotypes. Using this [*ha*^-/-^; Tg (β-actin:DsRed)] animals as the donors, transplantation experiments were conducted by the same way. The presence or absence of otoliths and the number of fluorescent cells were were assessed by observations around st. 29 using a confocal fluorescence microscope (Zeiss, LSM710). To evaluate how many *wt* cells were contained in the epithelium of the chimeric embryo, we calculated the ratio of area of the red labeled cells to all epithelial cells in a single plane of confocal images. For each otic vesicle, we select a plane where *wt* (red) cells are most abundant. ImageJ software was used for measuring the areas of transplanted cells (<http://rsbweb.nih.gov/ij/>).

**OlPKS expression in *A. oryzae* and medaka rescue assay.** The entire *olpks* ORF was amplified and subcloned into a fungal expression vector pTAex3. The resulting expression plasmid pTA-*olpks* was used for transformation of *Aspergillus oryzae* M-2-3 according to the protoplast-PEG method described by Gomi et al. [[12](#_ENREF_12)]. They were subcultured in minimal medium (Czapek-Dox) plate. The pTA-*olpks* transformans were inoculated into 10 mL preculture medium (carbon source: glucose 20 g/L) in a 50 mL tube (Falcon) and cultured at 30C for 3 days on a rotary shaker (150 rpm). Each 10 ml of the seed culture was inoculated into 1 L of induction medium (carbon source: starch 20g/L) for 4 days using the same cultural conditions. The mycelia from 1 L culture collected by filtration were added acetone (2 L) and stirred for 1 hour at room temperature to extract polyketide-derivatives. The acetone extract was filtered and the filtrate was concentrated to small volume to remove acetone. The residue (200 ml) was moved to a 2 L separating funnel and added 1 L H_2_O and 0.5 L ethyl acetate to partition between ethylacete/H_2_O (x 2). The ethyl acetate (upper) layer (1 L) was concentrated to dryness to give a colorless oil (approx. 0.5 g). The oil was next partitioned between hexane (200 ml)/90% methanol (200 ml), and 90% methanol layer (lower layer) was collected and concentrated to dryness to give the material (approx. 0.2 g) for medaka assay. The material was re-dissolved in a small amount of DMSO (approx. 1 ml) for the medaka assay. We prepared the negative control from a transformant with an empty pTAex3 vector.

Each extract re-dissolved in DMSO was diluted in 1:10-1:5 with Yamamoto’s Ringer solution. The chorions of *ha* embryos, stage of st. 21, were gently polished with a sandpaper to remove the attaching filaments, and incubated with the solution overnight. After brief washing by Yamamoto’s Ringer solution, embryos were grown in normal Yamamoto’s Ringer solution another night until evaluation. Around st. 29, we evaluated whether one otic vesicle had otoliths (either 1 or 2 otoliths in the otic vesicle) or didn’t have otolith at all.

**Purification of OlPKS expressed in *A.oryzae* transformant.** A transformant expressing OlPKS was cultivated as described above and mycelium was collected and frozen. Mycelium was ground in a frozen mortar by a pestle and suspended in buffer A (50 mM KH_2_PO_4_ [pH 7.5], 150 mM NaCl, 10% glycerol, 1 mM Tris[2-carboxyethyl]phosphine and Complete EDTA-free protease inhibitor cocktail (Roche)). The mixture was stirred at 4C for 15 min and subsequently centrifuged (12,000 g, 15 minutes). The supernatant was filtered through Miracloth (Calbiochem). Ni-NTA superflow (Qiagen) was added to the supernatant, and the solution was stirred at 4C for 1 hour. The protein/resin mixture was loaded into a gravity flow column, and proteins were purified with a gradient of imidazole in buffer A. The purified histidine-tagged protein was dialyzed against buffer A.

**Western blotting for purified OlPKS.** Purified OlPKS protein was mixed with 2x SDS buffer and shaken at 37C. After separation by 7.5% polyacrylamide gels, gels were transferred onto polyvinylidene difluoride membranes (Millipore) by semidry-blotting with three buffers (recommended by Atto (Atto, AE-1460 EzBlot)). Following procedure was performed as previously described [[13](#_ENREF_13)]. Anti-OlPKS serum was used for primary antibody (dilution is 1:20,000).

**Cloning of sea urchin *pks* genes.** Two *pks* genes have been reported for a sea urchin *Strongylocentrotus purpuratus*, *sppks-1* (XM_788471 [[14](#_ENREF_14)]) and *sppks-2* (NM_001252084 [[15](#_ENREF_15)]). Based on these sequences, primers were designed for the amplification of *H. pulcherrimus* *pks* genes. Mixed embryonic cDNA (gastrula, prism and pluteus stages) was used as the template for amplification using ExTaq (TaKaRa). The partial fragments of *hppks-1* (1146 nt) and *hppks-2* (1219 nt) were isolated by using following specific primers, respectively (5’ to 3’): CCTGTTTTCTAATCAGTGCTTG and ACRGATCCAATCTTGAGAGG, GATCGCTAACGCTCAACCAT and GTACCWGTACCATGYGCTTC.

**Whole mount *in situ* hybridization for sea urchin embryos.** Sea urchin (*Hemicentrotus pulcherrimus*) gametes were collected from adults and fertilized. Embryos were cultured in sea water until sampling. Embryos and larvae were fixed in 4% paraformaldehyde. Whole mount *in situ* hybridization was performed according to Arenas-Mena *et al.* [[16](#_ENREF_16)] with a slight modification, using a different blocking buffer (0.1 M Tris-Cl (pH7.5), 0.15 M NaCl, 0.5% Blocking Reagent (Roche)) for anti-DIG antibody incubation. Riboprobes containing digoxigenin-UTP were synthesized by standard methods. Antisense and sense (control) probes were made for partial sequences of *hppks-1* or *hppks-2*.

**Molpholino knockdown in sea urchin embryos.** Microinjection of MOs was carried out as previously described with some modifications [[17](#_ENREF_17)]. De-jellied unfertilized eggs were arrayed in row on a plastic dish coated with 1% of protamine sulfate (Wako). After insemination in filtered natural sea water containing 1 mM 3-amino-1H-1, 2, 4-triazole (Wako), microinjection was performed with micromanipulators (Narishige, MN-4 and MO-202U) and Femtojet (Eppendorf). The sequence of Standard MO (Gene Tools LLC) was follow: 5'-CCTCTTACCTCAGTTACAATTTATA-3'

**References**

1. Hojo M, Takashima S, Kobayashi D, Sumeragi A, Shimada A, Tsukahara T, Yokoi H, Narita T, Jindo T, Kage T, et al: **Right-elevated expression of charon is regulated by fluid flow in medaka Kupffer's vesicle.** *Development, growth & differentiation* 2007, **49:**395-405.

2. Koster R, Stick R, Loosli F, Wittbrodt J: **Medaka spalt acts as a target gene of hedgehog signaling.** *Development (Cambridge, England)* 1997, **124:**3147-3156.

3. Hochmann S, Aghaallaei N, Bajoghli B, Soroldoni D, Carl M, Czerny T: **Expression of marker genes during early ear development in medaka.** *Gene Expr Patterns* 2007, **7:**355-362.

4. Bajoghli B, Ramialison M, Aghaallaei N, Czerny T, Wittbrodt J: **Identification of starmaker-like in medaka as a putative target gene of Pax2 in the otic vesicle.** *Developmental dynamics : an official publication of the American Association of Anatomists* 2009, **238:**2860-2866.

5. Nemoto Y, Chatani M, Inohaya K, Hiraki Y, Kudo A: **Expression of marker genes during otolith development in medaka.** *Gene Expr Patterns* 2008, **8:**92-95.

6. Murayama E, Herbomel P, Kawakami A, Takeda H, Nagasawa H: **Otolith matrix proteins OMP-1 and Otolin-1 are necessary for normal otolith growth and their correct anchoring onto the sensory maculae.** *Mechanisms of development* 2005, **122:**791-803.

7. Kamura K, Kobayashi D, Uehara Y, Koshida S, Iijima N, Kudo A, Yokoyama T, Takeda H: **Pkd1l1 complexes with Pkd2 on motile cilia and functions to establish the left-right axis.** *Development (Cambridge, England)* 2011, **138:**1121-1129.

8. Stamatakis A, Hoover P, Rougemont J: **A rapid bootstrap algorithm for the RAxML Web servers.** *Systematic biology* 2008, **57:**758-771.

9. Castoe TA, Stephens T, Noonan BP, Calestani C: **A novel group of type I polyketide synthases (PKS) in animals and the complex phylogenomics of PKSs.** *Gene* 2007, **392:**47-58.

10. Kroken S, Glass NL, Taylor JW, Yoder OC, Turgeon BG: **Phylogenomic analysis of type I polyketide synthase genes in pathogenic and saprobic ascomycetes.** *Proceedings of the National Academy of Sciences of the United States of America* 2003, **100:**15670-15675.

11. Shimada A, Yabusaki M, Niwa H, Yokoi H, Hatta K, Kobayashi D, Takeda H: **Maternal-zygotic medaka mutants for fgfr1 reveal its essential role in the migration of the axial mesoderm but not the lateral mesoderm.** *Development (Cambridge, England)* 2008, **135:**281-290.

12. Gomi K, Iimura Y, Hara S: **Integrative Transformation of Aspergillus oryzae with a Plasmid Containing the Aspergillus nidulans argB Gene.** *Agric Biol Chem* 1987, **51:**2549-2555.

13. Matsuo M, Shimada A, Koshida S, Saga Y, Takeda H: **The establishment of rotational polarity in the airway and ependymal cilia: analysis with a novel cilium motility mutant mouse.** *Am J Physiol Lung Cell Mol Physiol* 2013, **304:**736-745.

14. Calestani C, Rast JP, Davidson EH: **Isolation of pigment cell specific genes in the sea urchin embryo by differential macroarray screening.** *Development (Cambridge, England)* 2003, **130:**4587-4596.

15. Beeble A, Calestani C: **Expression pattern of polyketide synthase-2 during sea urchin development.** *Gene Expr Patterns* 2012, **12:**7-10.

16. Arenas-Mena C, Cameron AR, Davidson EH: **Spatial expression of Hox cluster genes in the ontogeny of a sea urchin.** *Development (Cambridge, England)* 2000, **127:**4631-4643.

17. Katow H, Katow T, Abe K, Ooka S, Kiyomoto M, Hamanaka G: **Mesomere-derived glutamate decarboxylase-expressing blastocoelar mesenchyme cells of sea urchin larvae.** *Biology open* 2014, **3:**94-102.
