# Supplementary material for: Unexpected link between polyketide synthase and calcium carbonate biomineralization
Source: Zoological Lett. 2015 Jan 13;1:3. doi: 10.1186/s40851-014-0001-0 (PMC4604110; doi:10.1186/s40851-014-0001-0)
Supplement: Additional file 2: — Phenotypic analyses in ha mutant. (A-F) Whole-mount in situ hybridization of molecular markers expressed in the OVs. (A)(B) Differentiation markers, eya-1 (across the vesicles) and pax-2 (medial region) are normally expressed in ha embryos. All OVs are dorsal views. (C)(D) Normal expression patterns of marker genes. dlx-3b is restricted to the dorsal wall of the OV and bmp-4 marks the neural cristae regions. ac,anterior cristae; lc, lateral cristae; pc, posterior cristae. All OVs are lateral views. Left side shows anterior. (E)(F) The transcripts of otolith matrix proteins, starmaker-like and sparc-1 (are comparably detected in the mutant OV. All OVs are dorsal views. (G) TEM images from a wt specimen and a mutant one (representative images are shown in G to G’’’). Asterisks: growing otolith precursors; Arrows: fine substances; ‘s’: seeding particles; ‘g’: globules. (H) Immunohistochemistry for OMP-1 revealed crystalized ‘otolith’ of ha in hatching stage (st. 40) that looks similar to wt otolith and contains at least some organic materials. (I) double colored-Immunofluorescence of acetylated α-tubulin and γ-tubulin (root of cilia). Short cilia (<1μm) are visible in the OV of ha as well as wt. (J) Scatter plot showing total number of cilia in the OV of wt and ha at various stages. Total number of cilia in the OV is obtained by counting the signal of α-tubulin and γ-butulin. In each stage, there was no significant difference in total number of cilia between wt and ha (>0.05 Student’s t-test). (K) Otolith development in the OV of ktu mutant. In ktu, seeding particles are coalesced at the prospective macula region (Upper; dorsal views of left OV), and otoliths appear normal at later stage just like wt (Middle; dorsal views of left OV and Lower; lateral views of left OV). Asterisks: otoliths, Arrow: paste-like substance. [file 40851_2014_1_MOESM2_ESM.pdf]

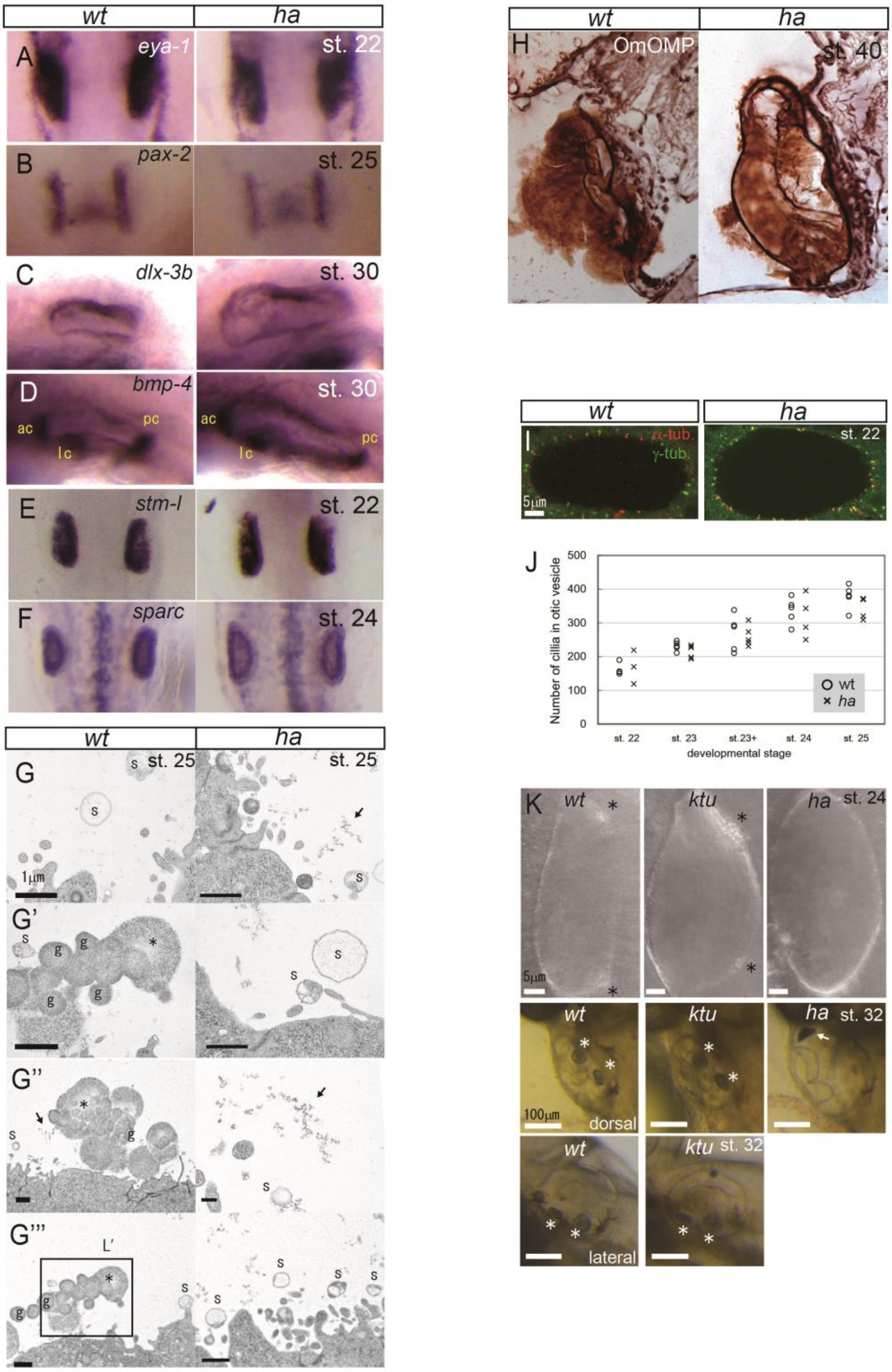

Additional file 1

Additional file 1.

Phenotypic analyses in *ha* mutant. (A-F) Whole-mount *in situ* hybridization of molecular markers expressed in the OVs. (A)(B) Differentiation markers, *eya-1* (across the vesicles) and *pax-2* (medial region) are normally expressed in *ha* embryos. All OVs are dorsal views. (C)(D) Normal expression patterns of marker genes at stage st. 30. *dlx-3b* is restricted to the dorsal wall of the OV and *bmp-4* marks the neural cristae regions, showing that regionalization and neural differentiation patterns are not disturbed in *ha* mutant. Mutant OVs are enlarged. ac,anterior cristae; lc, lateral cristae; pc, posterior cristae. All OVs are lateral views. Left side shows anterior. (E)(F) The transcripts of otolith matrix proteins, *starmaker-like* (st. 22) and *sparc-1* (st. 24) are comparably detected in the mutant OV. All OVs are dorsal views. Mutant OVs are enlarged at st. 24. (G) TEM images from a *wt* specimen and a mutant one (representative images are shown in G to G'''). Asterisks: growing otolith precursors; Arrows: fine substances; 's': seeding particles; 'g': globules. (H) Immunohistochemistry for OMP-1 revealed crystalized 'otolith' of *ha* in hatching stage (st. 40) that looks similar to *wt* otolith and contains at least some organic materials. (I) double colored-Immunofluorescence of acetylated α-tubulin and γ-tubulin (root of cilia). Many short cilia (<1μm) are visible in the OV of *ha* as well as *wt*. (J) Scatter plot showing total number of cilia in the OV of *wt* and *ha* at various stages. Total number of cilia in the OV is obtained by counting the signal of α-tubulin (cilia) and γ-butulin (root of cilia). In each stage, there was no significant difference in total number of cilia between *wt* and *ha* (>0.05 Student's t-test). (K) Otolith development in the OV of *ktu* mutant. In *ktu*, seeding particles are coalesced at the prospective macula region (*Upper*; dorsal views of left OV), and otoliths appear normal at later stage just like *wt* (*Middle*; dorsal views of left OV and *Lower;* lateral views of left OV). Asterisks: otoliths, Arrow: paste-like substance
