# Supplementary material for: Unexpected link between polyketide synthase and calcium carbonate biomineralization
Source: Zoological Lett. 2015 Jan 13;1:3. doi: 10.1186/s40851-014-0001-0 (PMC4604110; doi:10.1186/s40851-014-0001-0)
Supplement: Additional file 5: — Mapping analyses and expression pattern of the ha gene. (A) Sequencing of the ha gene in two mutant fish. ki79 fish harbors a nonsense mutation at 3856 nt of the ORF2 locus. (B) Architecture of OlPKS protein predicted from the amino acid sequence (Upper). Critical residues of each domain used for the mRNA injection experiment are shown by arrows. Mutation site of each mutant is shown by arrowhead. Three amino acids corresponding to 9-bp deletion is ‘KPS’. Central proline is found to be a highly conserved residue among PKSs although it is not previously considered as a conserved motif. Based on the structure of mammal fatty acid synthase (FAS), it is possible that the proline contribute to help a substrate to enter the active site of KS. The red bar indicates the region used as an antigen for producing anti-serum of OlPKS. Conserved motifs found in the amino acid sequence of OlPKS (Lower table). Asterisks show residues mutated in mRNA rescue experiments. Underlines show conserved amino acid residues. (C) Whole-mount in situ hybridization with olpks probes at st. 22. The dorsally- and medially- restricted pattern is evidenced by lateral view (Upper) and histology of the OV region (Lower). Yellow dashed lines show OVs. (D) Expression profiles of olpks and paralogous gene candidates in the embryonic stages or adult tissues. A RT-PCR analysis reveals that olpks is expressed but olpks-2 and olpks-3 are not expressed and that olpks is expressed only around embryonic stage 22. [file 40851_2014_1_MOESM5_ESM.pdf]

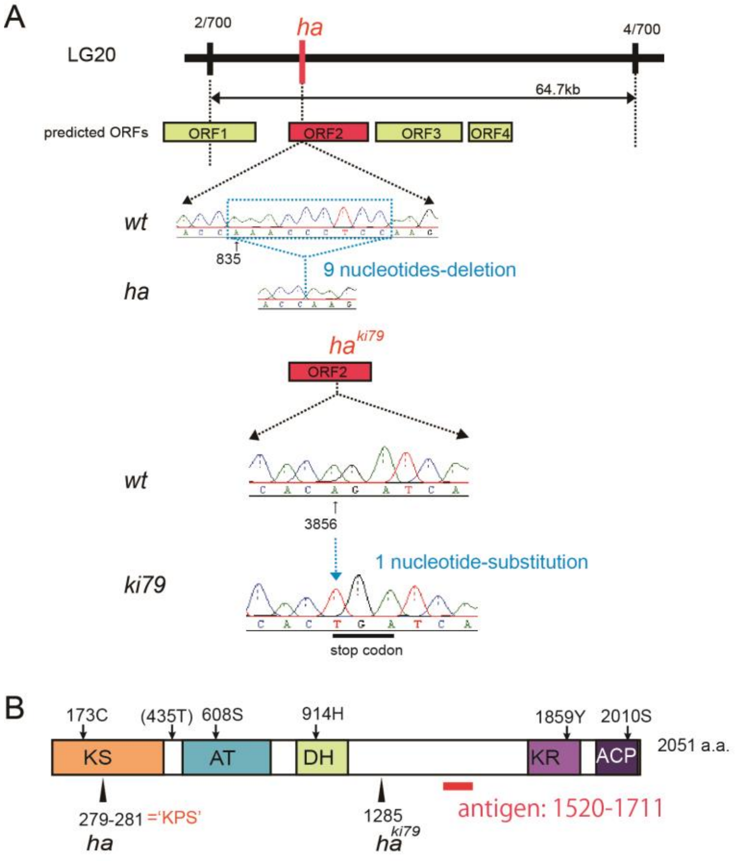

Name	Ketoacyl Synthase	Acyl Transferase	Dehydratase	Ketoacyl Reductase	Acyl Carrier Protein
Abbreviation	KS	AT	DH	KR	ACP
Amino acid sequence of OIPKS	*CSSS (173-176)<br>HGTGT (305-109)<br>NIGH (343-346)	GHS*IG (606-610)<br>YHSH (714-717)	*HKNNGVAIVP (914-923)<br>DYEMQ (1078-1082)	TGGISGLG (1714-1721)<br>*YAAA (1859-1862)	DS*ML (2009-2012)

asterisks: residues mutated in rescue experiments
underlines: conserved amino acid residues

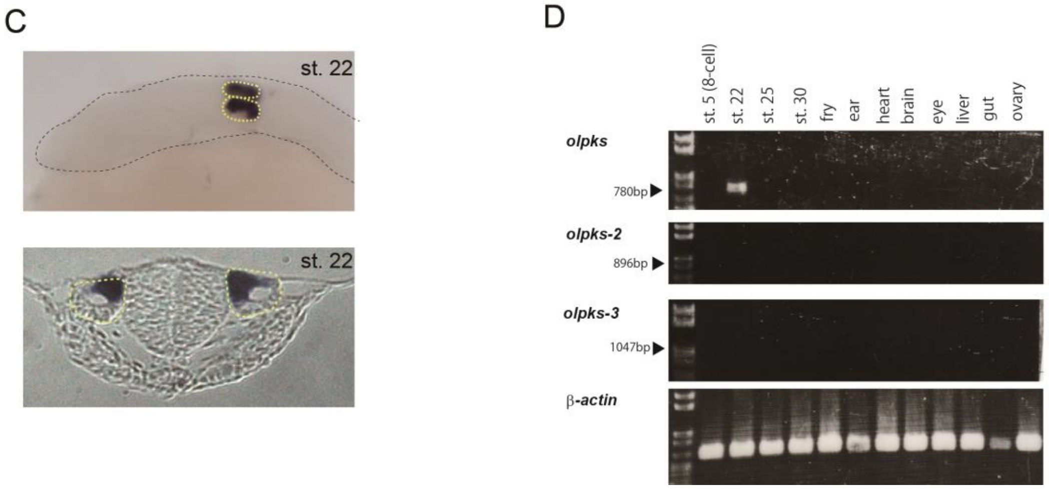

Additional file 2.

Mapping analyses and expression pattern of the *ha* gene. (A) Sequencing of the *ha* gene in two mutant fish. *ki79* fish harbors a nonsense mutation at 3856 nt of the ORF2 locus. (B) Architecture of OlPKS protein predicted from the amino acid sequence (*Upper*). Critical residues of each domain used for the mRNA injection experiment are shown by arrows. Mutation site of each mutant is shown by arrowhead. Three amino acids corresponding to 9-bp deletion is 'KPS'. Central proline is found to be a highly conserved residue among PKSs although it is not previously considered as a conserved motif. Based on the structure of mammal fatty acid synthase (FAS), it is possible that the proline contribute to help a substrate to enter the active site of KS. The red bar indicates the region used as an antigen for producing anti-serum of OlPKS. Conserved motifs found in the amino acid sequence of OlPKS (*Lower table*). Asterisks show residues mutated in mRNA rescue experiments. Underlines show conserved amino acid residues. (C) Whole-mount *in situ* hybridization with *olpks* probes at st. 22. The dorsally- and medially- restricted pattern is evidenced by lateral view (*Upper*) and histology of the OV region (*Lower*). Yellow dashed lines show OVs. (D) Expression profiles of *olpks* and paralogous gene candidates in the embryonic stages or adult tissues. A RT-PCR analysis reveals that *olpks* is expressed but *olpks-2* and *olpks-3* are not expressed and that *olpks* is expressed only around embryonic stage 22.
