# Supplementary material for: Unexpected link between polyketide synthase and calcium carbonate biomineralization
Source: Zoological Lett. 2015 Jan 13;1:3. doi: 10.1186/s40851-014-0001-0 (PMC4604110; doi:10.1186/s40851-014-0001-0)
Supplement: Additional file 6: — Analyses of property of the OlPKS product. (A)(B) Imaging analyses of the chimeric experiment. (A) All 110 OVs of 55 transplanted animals are observed and sorted to 9 categories depending on their percentage of wt cells in their OV epithelium (Note a chimeric fish has two OVs). The histogram shows that a very small number of wt cells can rescue otolith formation. (B) The same sample set is also analyzed to assess the localization of wt cells within the OV. ‘medial’ means wt cells located around medial wall of the OV region where olpks gene is normally expressed in wt animal; ‘medial and other’ includes medial and any other epithelial region; ‘other’ includes wt cells existing anywhere in the epithelium except the medial wall. (C) Western blot of OlPKS protein produced by A. oryzae. To verify expression of OlPKS in heterologous expression experiment, purified protein from a transformant is analyzed by anti-OlPKS antiserum. The OlPKS protein gave a protein band at a position of ca. 210 kDa on SDS-PAGE. However, some bands (degradade proteins) were also found in this experiment. [file 40851_2014_1_MOESM6_ESM.pdf]

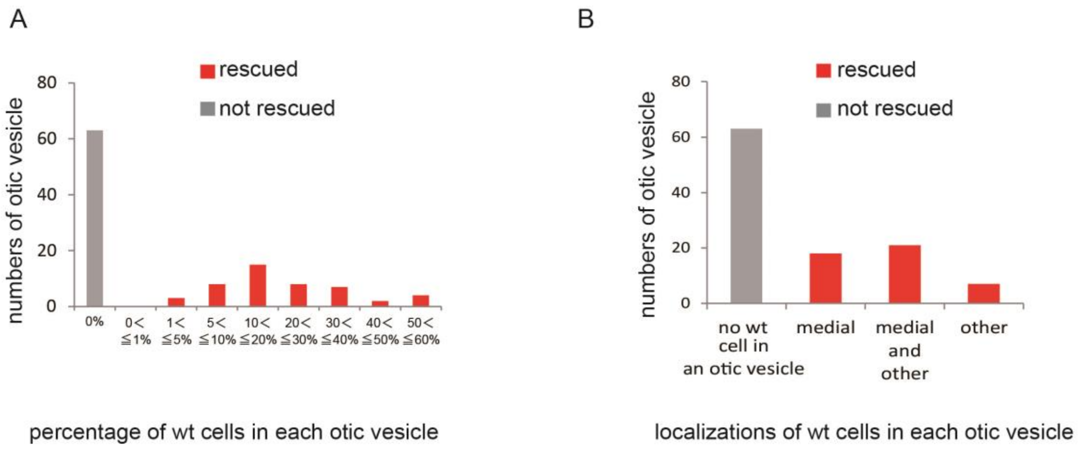

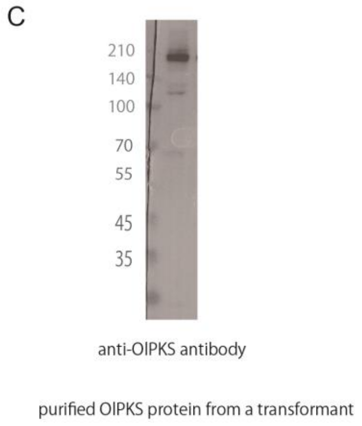

Additional file 3

Analyses of property of the OlPKS product. (A)(B) Imaging analyses of the chimeric experiment. (A) All 110 OVs of 55 transplanted animals are observed and sorted to 9 categories depending on their percentage of *wt* cells in their OV epithelium (Note a chimeric fish has two OVs). The histogram shows that a very small number of *wt* cells can rescue otolith formation. (B) The same sample set is also analyzed to assess the localization of *wt* cells within the OV. 'medial' means *wt* cells located around medial wall of the OV region where *olpks* gene is normally expressed in *wt* animal; 'medial and other' includes medial and any other epithelial region; 'other' includes *wt* cells existing anywhere in the epithelium except the medial wall. (C) Western blot of OlPKS protein produced by *A. oryzae*. To verify expression of OlPKS in heterologous expression experiment, purified protein from a transformant is analyzed by anti-OlPKS antiserum. The OlPKS protein gave a protein band at a position of ca. 210 kDa on SDS-PAGE. However, some bands (degradade proteins) were also found in this experiment.
