# Supplementary material for: Unexpected link between polyketide synthase and calcium carbonate biomineralization
Source: Zoological Lett. 2015 Jan 13;1:3. doi: 10.1186/s40851-014-0001-0 (PMC4604110; doi:10.1186/s40851-014-0001-0)
Supplement: Additional file 7: — Broad distribution of animal PKSs. (A) Maximum parsimony phylogenetic estimate of relationships among animal type I PKSs and FASs, based on an alignment of amino acid sequences of the KS domain. Animal PKSs containing OlPKS are not divided from other bacterial or fungal PKSs, as indicated by low values of the bootstrap. By contrast, the animal FAS clade is clearly separated from animal PKSs. Protein accession number of Genbank or other database is shown with species name. Numbers described around branches indicate percentage of the bootstrap value supporting each clade. Branch length indicates number of inferred amino acid changes. (B) Representative image of whole-mount in situ hybridization of zebrafish embryo using probes for pks homologue. The transcript of a pks homologue, drpks(wu:fc01d11), is exclusively expressed in zebrafish OV at the 20 somite-stage (19hpf). Arrows: OVs. (C) Summary of knockdown experiment using MOs. Injected embryos are categorized by the phenotype of spicule. HpPKS-1 knockdown animal lacked pigment while HpPKS-1 control MO (CMO)-injected animals showed normal pigmentation. These two morphants didn’t show any spicule abnormality. Injection of HpPKS-1 MO and HpPKS-1 CMO at high concentration (200 μM) caused developmental arrest at the gastrulation. Various concentrations of HpPKS-2 MO injection (three levels; 50, 100 and 200 μM) shows dose dependency. All HpPKS-2 CMO-injected embryos showed no spicule defect. Standard control MO did not give any effect. [file 40851_2014_1_MOESM7_ESM.pdf]

A

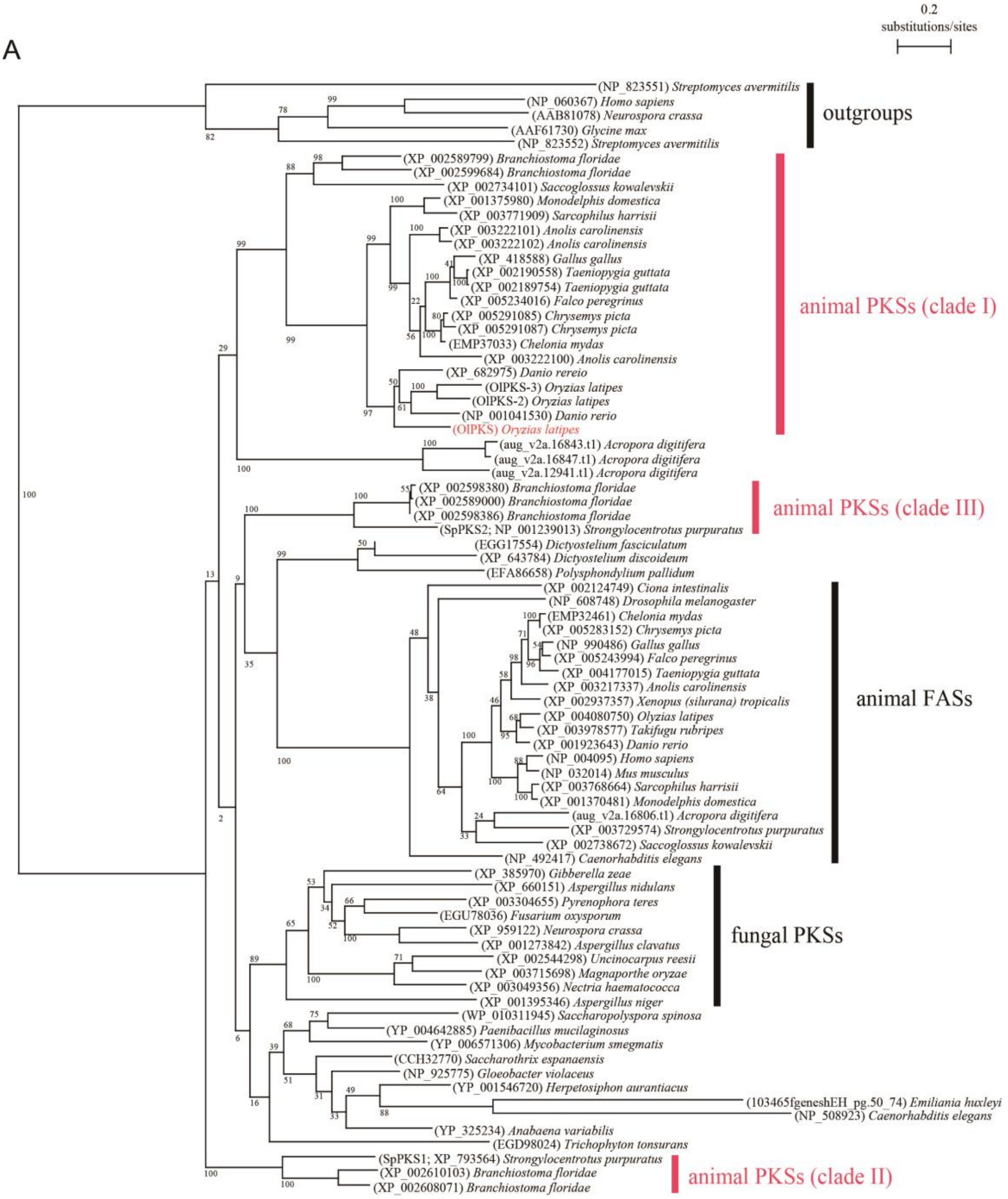

B

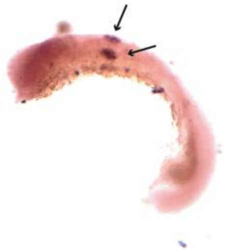

C

MO		fully or partially crystalized spicule	no spicule	developmental arrest and necrotic death	total	note
HpPKS-1	100uM	109	0	6	115	no pigmentation
	200uM	114	0	264	378	no pigmentation
HpPKS-1 Control	200uM	246	0	55	301	
HpPKS-2	50uM	61	79	0	140	
	100uM	5	149	0	154	
	200uM	10	477	0	487	
HpPKS-2 Control	200uM	288	0	1	289	
Standard Control	200uM	237	0	8	245	

Additional file 4.

Broad distribution of animal PKSs. (A) Maximum parsimony phylogenetic estimate of relationships among animal type I PKSs and FASs, based on an alignment of amino acid sequences of the KS domain. Animal PKSs containing OlPKS are not divided from other bacterial or fungal PKSs, as indicated by low values of the bootstrap. By contrast, the animal FAS clade is clearly separated from animal PKSs. Protein accession number of Genbank or other database is shown with species name. Numbers described around branches indicate percentage of the bootstrap value supporting each clade. Branch length indicates number of inferred amino acid changes. (B) Representative image of whole-mount *in situ* hybridization of zebrafish embryo using probes for *pks* homologue. The transcript of a *pks* homologue, *drpks(wu:fc01d11)*, is exclusively expressed in zebrafish OV at the 20 somite-stage (19hpf). Arrows: OVs. (C) Summary of knockdown experiment using MOs. Injected embryos are categorized by the phenotype of spicule. HpPKS-1 knockdown animal lacked pigment while HpPKS-1 control MO (CMO)-injected animals showed normal pigmentation. These two morphants didn't show any spicule abnormality. Injection of HpPKS-1 MO and HpPKS-1 CMO at high concentration (200 μM) caused developmental arrest at the gastrulation. Various concentrations of HpPKS-2 MO injection (three levels; 50, 100 and 200 μM) shows dose dependency. All HpPKS-2 CMO-injected embryos showed no spicule defect. Standard control MO did not give any effect.
